# Evaluation in Monogenic Diabetes of the Impact of *GCK*, *HNF1A*, and *HNF4A* Variants on Splicing through the Combined Use of In Silico Tools and Minigene Assays

**DOI:** 10.1155/2023/6661013

**Published:** 2023-08-31

**Authors:** Delphine Bouvet, Amélie Blondel, Jean-Madeleine de Sainte Agathe, Gwendoline Leroy, Cécile Saint-Martin, Christine Bellanné-Chantelot

**Affiliations:** Department of Medical Genetics, AP-HP Pitié-Salpêtrière Hospital, Sorbonne University, Paris, France

## Abstract

Variants in *GCK*, *HNF1A*, and *HNF4A* genes are the three main causes of monogenic diabetes. Determining the molecular etiology is essential for patients with monogenic diabetes to benefit from the most appropriate treatment. The increasing number of variants of unknown significance (VUS) is a major issue in genetic diagnosis, and assessing the impact of variants on RNA splicing is challenging, particularly for genes expressed in tissues not easily accessible as in monogenic diabetes. The in vitro functional splicing assay based on a minigene construct is an appropriate approach. Here, we performed in silico analysis using SpliceAI and SPiP and prioritized 36 spliceogenic variants in *GCK*, *HNF1A*, and *HNF4A*. Predictions were secondarily compared with Pangolin and AbSplice-DNA bioinformatics tools which include tissue-specific annotations. We assessed the effect of selected variants on RNA splicing using minigene assays. These assays validated splicing defects for 33 out of 36 spliceogenic variants consisting of exon skipping (15%), exonic deletions (18%), intronic retentions (24%), and complex splicing patterns (42%). This provided additional evidence to reclassify 23 out of 31 (74%) VUS including missense, synonymous, and intronic noncanonical splice site variants as likely pathogenic variants. Comparison of in silico analysis with minigene results showed the robustness of bioinformatics tools to prioritize spliceogenic variants, but revealed inconsistencies in the location of cryptic splice sites underlying the importance of confirming predicted splicing alterations with functional splicing assays. Our study underlines the feasibility and the benefits of implementing minigene-splicing assays in the genetic testing of monogenic diabetes after a prior in-depth in silico analysis.

## 1. Introduction

Maturity-onset diabetes of the young (MODY) is an autosomal dominant form of monogenic diabetes characterized by clinical and genetic heterogeneity. Identifying the genetic etiology is important for patients with MODY to benefit from the most effective treatment and the clinical care of associated diseases and for genetic counseling [1, 2]. Disease-causing variants in the glucokinase gene (*GCK*), hepatocyte nuclear factor 1A (*HNF1A*), and *HNF4A* account for about 75% of MODY diagnoses [3, 4]. All are responsible for a primary abnormal insulin secretion. The glucokinase acts as a glucose sensor in the pancreatic beta cells. HNF1A and HNF4A are transcription factors involved in beta cell development and in the regulation of both pancreatic and hepatic genes. Several hundred variants have been reported in these three major causes, and most of them are identified in single families [5, 6]. Novel variants are still frequently identified, and their disease causality remains challenging in the absence of easy-to-implement functional characterization. We and others have estimated that about 15% to 40% of novel variants identified in *GCK*, *HNF1A*, and *HNF4A* variants are of unknown significance (VUS) (see [7] and the ClinVar database).

These VUS are either exonic variants (missense and synonymous) or intronic variants located in noncanonical splice site sequences. Some of them could impact RNA splicing by abolishing or decreasing the recognition of the physiological 3′ and 5′ splice sites (3′ss and 5′ss, respectively), creating de novo splice sites, activating preexisting cryptic splice sites, or modifying splicing regulatory sequences [8]. The resulting splicing alterations commonly lead to exon skipping, partial exonic deletion, or intronic retention that could be pathogenic [9].

While splicing defects are increasingly associated with inherited diseases [10, 11], very few spliceogenic variants located outside canonical splice sites have been validated in MODY [12–17]. This is probably because the genes involved in MODY are mostly expressed in the pancreas and liver, two tissues not easily accessible, thus making it difficult to assess the effects of a variant on in vivo RNA splicing. In this context, the in vitro functional splicing assay based on a minigene construct is an appropriate approach [18]. However, prior in silico analysis is important to prioritize potential splice-altering variants for testing by minigene assays and to integrate this approach in genetic testing.

Herein, we investigated whether synonymous, missense, and intronic noncanonical splice-site variants in *GCK*, *HNF1A*, and *HNF4A* could result in splicing alterations. We prioritized 36 variants based on in silico predictions and analyzed them using minigene assays. Thirty-three variants were found to impact RNA splicing which provided additional evidence for their pathogenicity. Thus, 24 VUS could be reclassified as likely pathogenic after minigene assays and led to a definitive diagnostic of monogenic diabetes.

## 2. Materials and Methods

### 2.1. Editorial Policies and Ethical Considerations

Ethical committee approval was not required for this study given that all experiments were performed in vitro by transient transfection of constructs into cultured cells. The DNA collection of the Department of Medical Genetics of the Pitié-Salpêtrière Hospital has been declared to the minister for research and the director of the regional health agency (Biobank ID #DC2009-957). Patients signed an informed consent for genetic testing and for any research project performed in relation with their disease. Results of the genetic analyses were registered in an internal restricted-access diagnosis database (CNIL certificate 16/02/2010-n°1412729).

### 2.2. Variant Selection

We extracted from our in-house MODY diagnosis database the *GCK*, *HNF1A*, and *HNF4A* variants classified as variant of unknown significance (VUS), likely pathogenic (LP), or pathogenic (P) following the American College of Medical Genetics and Genomics guidelines [19]. The following reference transcripts were used: *GCK* (NM_000162.5), *HNF1A* (NM_000545.6), and *HNF4A* (NM_175914.4).

**Figure 1:**
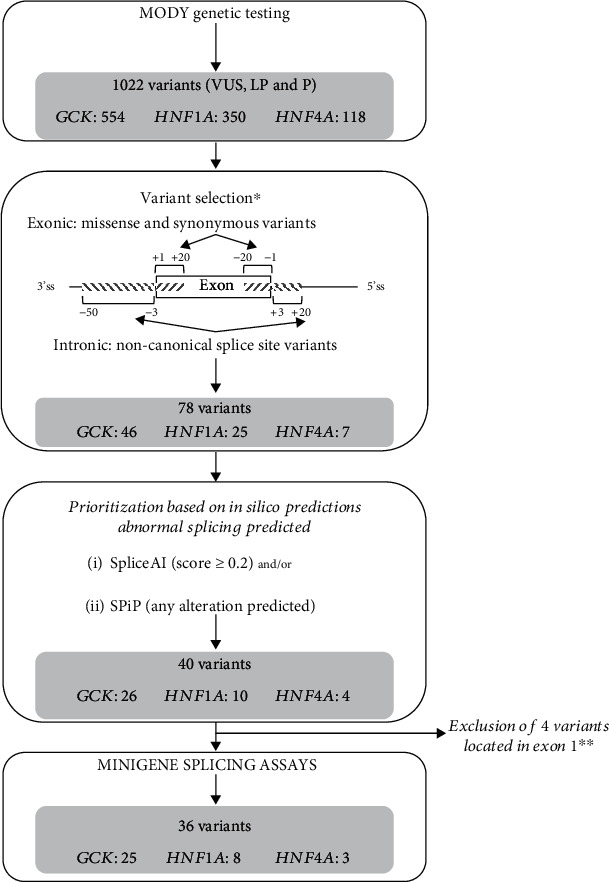
Variant selection for minigene assays. This flowchart shows the sequential steps of variant selection. ^∗^Exclusion of canonic splice sites (-1 and -2; +1 and +2), nonsense, and frameshift variants. ^∗∗^Four variants located in the exon 1 requiring specific plasmid construction were excluded. In our series, no variant was located in the last exons. VUS: variant of unknown significance; LP: likely pathogenic; P: pathogenic.

We then selected variants located between the positions -50 to +20 of the 3′ splice site (ss) and -20 to +20 of the 5′ss. Nonsense, frameshifts, and intronic variants affecting canonical splice site positions (-1 and -2 of the 3′ss and +1 and +2 of the 5′ss) were excluded.  Figure 1 shows the sequential steps of variant selection.

### 2.3. In Silico Predictions of Splicing Alterations

The impact of the variant on RNA splicing was predicted by using two in silico algorithms available via the online application MobiDetails [20]: (i) SpliceAI 1.3 which is a computational tool based on a neural network [21]. We ran SpliceAI 1.3 using 500 bp nucleotides of flanking sequences as input. As recommended by Jaganathan et al. [21], a delta score (DS) threshold of 0.2 was chosen to consider a splicing alteration. We also used SpliceAI-visual bedGraphs [22] available via MobiDetails to get the raw scores of all predicted splice sites in the region of interest. (ii) SPiP 2.1 is a splicing prediction pipeline which includes complementary tools to evaluate the impact of the variant on different splicing motifs [23]. We considered all variants for which a splicing alteration was predicted by SPiP (regardless of the score).

In a second step, we compared splicing predictions of SpliceAI and SPiP with those of two recently developed bioinformatics tools that predict splicing in different tissues: (i) Pangolin (v1.0.2) based on a neural network and using four tissues [24]. A splice score ≥ 0.2 was chosen to consider a splicing alteration. (ii) AbSplice-DNA (v.1.0.0) uses a tissue-specific splicing annotation based on the Genotype-Tissue Expression (GTEx) project which maps acceptor and donor splices and quantifies their usage in 49 human tissues [25]. AbSplice-DNA scores were obtained from the precomputed AbSplice_DNA_hg38_single nucleotide variant site file. For each variant, only the maximal score across all the tissues was taken, and a prediction threshold > 0.01 was chosen [25].

### 2.4. Cell-Based Minigene-Splicing Assays

We performed splicing assays by using the two-exon minigene pCAS2 vector, as previously described [26]. Analyses of variants located in the first and last exons requiring specific minigene constructs were excluded from the study (Figure 1). All mutant constructs were confirmed by sequencing. The assay is based on the comparative analysis of the splicing pattern of the reference versus mutant minigenes transiently transfected into HeLa cells. Minigene transcripts were analyzed by RT-PCR, visualized on agarose gels, and sequenced. Primers used for the minigene assays are described in Supplemental Table 1.

### 2.5. Annotation of Splicing Alterations

Splicing alterations were annotated as follows: △ for exonic deletion, ▼ for intronic retention, p for the alteration of the 3′ss, and q for the alteration of the 5′ss. The number indicates the number of nucleotides inserted or deleted.

## 3. Results

### 3.1. Variant Selection and Prioritization Based on In Silico Predictions of Splicing Alterations

We focused our study on missense and synonymous variants together with intronic noncanonical splice site variants of *GCK*, *HNF1A*, and *HNF4A*. We selected 78 variants (46 on *GCK*, 25 on *HNF1A*, and 7 on *HNF4A*) located in the regions -50 to +20 bp of the 3′ss and -20 to +20 bp of the 5′ss among 1022 variants identified in these three genes in the context of a MODY molecular diagnosis (Figure 1).

Next, in order to prioritize the variants to be tested with minigene-splicing assays, we assessed their potential impact on splicing by using two bioinformatics tools, SpliceAI and SPiP, and selected the variants predicted by at least one of the two tools to alter splicing. Hence, 40 variants were prioritized, and 36 (25 on *GCK*, 8 on *HNF1A*, and 3 on *HNF4A*) of them were analyzed, four being excluded due to their location in the first exon. Among those 36 variants, 32 (89%) were predicted by both tools (Table 1 and Supplemental Table 2) to impact RNA splicing.

### 3.2. RNA-Splicing Defects Detected by Minigene-Splicing Assays

**Figure 2:**
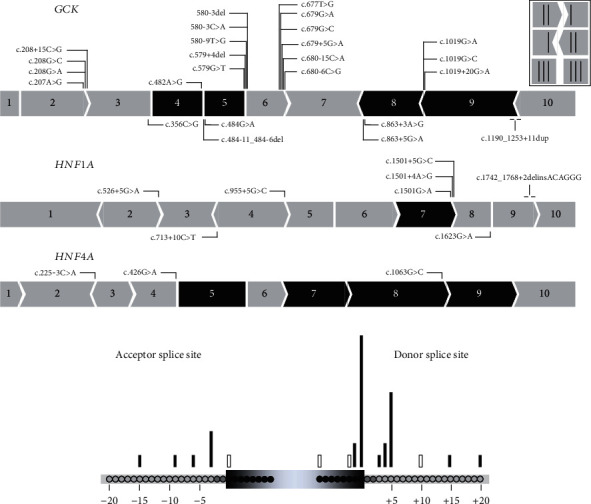
Overview of the 36 spliceogenic variants tested by minigene assays. (a) Location of the variants on *GCK* (*N* = 25), *HNF1A* (*N* = 8), and *HNF4A* (*N* = 3). The figure shows the schematic representation of the three genes. Exons indicated in black are exons whose splicing is in-frame. (b) Position of the tested spliceogenic variants relative to the acceptor and donor splice sites. Black bar: variants inducing splicing alterations; white bar: variants maintaining normal splicing in the minigene assay.

We performed minigene-splicing assays for the 36 prioritized variants consisting of 16 exonic variants (4 synonymous and 12 missense) located at exon termini up to 8 bp from splice sites, 18 intronic variants located between -3 to -15 bp of the 3′ss and +3 to +20 bp of the 5′ss, and 2 variants encompassing an exon-intron junction (Table 1 and Figure 2). Most variants (28/36, 78%) were located in the vicinity of the 5′ss. None of our variants were located on the branch site.

**Figure 3:**
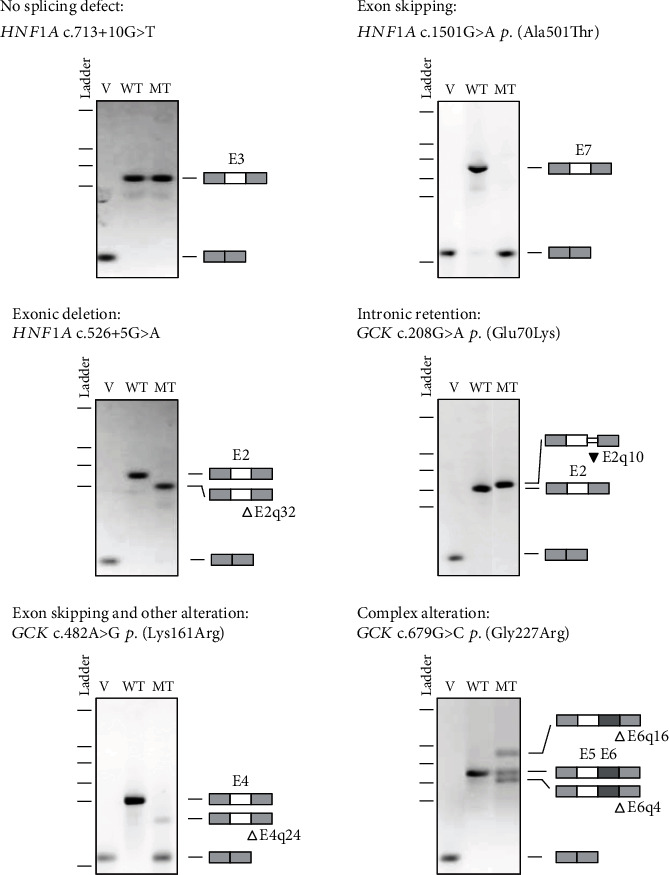
Illustration of each type of splicing effect based on minigene-splicing assay. The pictures show RT-PCR products visualized on agarose gels and annotated as described in Materials and Methods. v: empty pCAS2 vector; WT: wild type; MT: mutant. △: nucleotide deletion; ▼: nucleotide insertion.

For each type of splicing alteration (no splicing defect, exon skipping, exonic deletion, intronic retention, association of exon skipping and another minor alteration, and complex alterations), an example of the result is given in Figure 3. Results for the 36 variants are displayed on Supplemental Figure 1.

As shown in Supplemental Figure 1A, the minigene assays did not reveal any splicing alteration for three variants. Two of them had been predicted as spliceogenic by SPiP alone (Table 1 and Supplemental Table 2), including (i) the *GCK* missense variant (c.484G>A) predicted to alter the consensus 3′ss with a SPiP score of 0.304 and (ii) the intronic *HNF1A* variant (c.713+10C>T) predicted to activate a cryptic splice site (c.713+8) with a SPiP score of 0.43. For both variants, two independent minigene assays and sequencing of RT-PCR products did not show the splicing alterations predicted by SPiP (Supplemental Figure 2). The *GCK* missense variant (c.356C>G) was predicted by both algorithms to create a de novo donor splice site at position c.355. However, both SpliceAI and SPiP predict that the score of this cryptic splice site is lower than the score of the physiological splice site in the variant context (Supplemental Figure 2). This cryptic splice site (which induces the *Δ*E3q8 alteration) was shown to be used in the minigene assay, but the resulting transcript was observed at a very low level and considered insignificant (Supplemental Figure 2).

**Figure 4:**
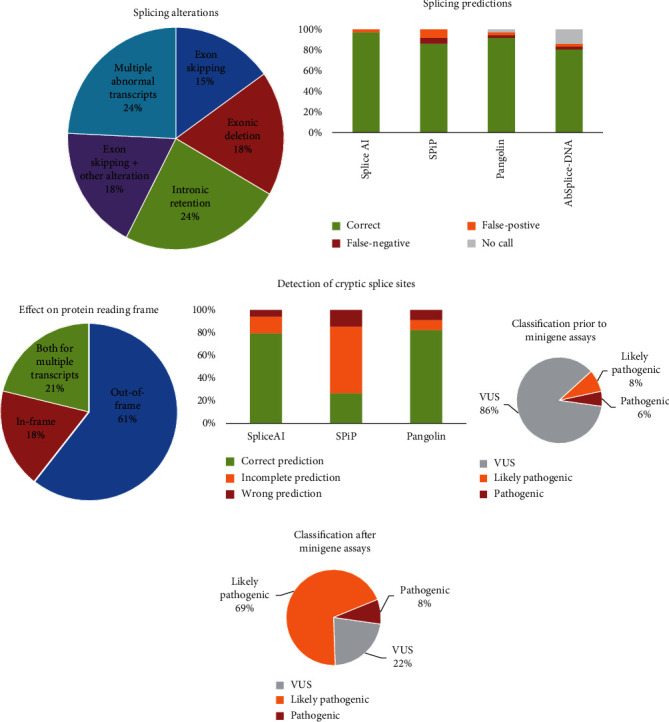
Characterization of splicing alterations, accuracy of in silico predictions, and impact on variant classification. (a) Distribution of splicing alterations. (b) Accuracy of in silico splicing predictions. (c) Impact of splicing defects on protein reading frame. (d) Accuracy of in silico tools to detect cryptic splice sites. An “incomplete prediction” was applied to variants for which a splicing alteration was predicted but the corresponding algorithm has not provided the position of the alternative splice site. Positions of cryptic splice sites were unavailable for AbSplice-DNA. Variant classification before (e) and after (f) minigene-splicing assays.

For 33 (91.7%) of the 36 variants tested, the minigene assays showed splicing alterations consisting of exon skipping (15%), exonic deletions (18%), intronic retentions (24%), cooccurrence of exon skipping and another alteration (18%), and complex splicing patterns (24%) (Table 1, Supplemental Table 2, Supplemental Figure 1B–1F, and Figure 4(a)).

Five variants (1 missense, 1 synonymous variant, 1 indel encompassing a splice site, and 2 small intronic deletions) led to exon skipping with a total loss of the corresponding full-length minigene transcripts (Supplemental Figure 1B). For the c.1623G>A *HNF1A* variant, SpliceAI did not predict an alteration of the consensus splice site and predicted the creation of a cryptic splice site in position c.1623+5 but with a raw score lower than that of the consensus splice site. SPiP predicted a decreased consensual splice site score, but still higher than the score of the cryptic splice site (Supplemental Figure 2). As the physiological splice site was not predicted to be altered, the exon skipping observed in the minigene assay was unexpected.

Six variants (1 missense, 1 synonymous, and 4 intronic), all located in the vicinity of the 5′ss, induced deletions of 2 to 32 bp of exonic sequences (Supplemental Figure 1C). For the *GCK* c.863+3A>G variant, only a loss of the natural donor splice site was predicted by SpliceAI, so one could assume at first sight an exon skipping as the most probable splice impact. However, the SpliceAI-visual bedGraph showed an upstream cryptic splice site at position c.853 whose raw score was not significantly altered in the variant context (0.99 versus 0.95 in the wild-type condition) but was greater than that of the altered natural splice site (0.75), suggesting this alternative splice site could be used. This hypothesis was the one confirmed in the minigene assay (Supplemental Figure 2). Among the six variants resulting in exonic deletion, three induced partial splicing defects as some proportion of the full-length transcripts was observed (Table 1, Supplemental Table 3, and Supplemental Figure 1C).

Seven variants led to intronic retention of 5 bp to 17 bp, and an additional variant (c.1190_1253+11dup) extended the exonic sequence via a 75 bp duplication (Table 1, Supplemental Figure 1D, and Supplemental Table 2). For the *HNF4A* c.225-3C>A variant, SpliceAI predicted the loss of the natural splice site (raw score in mutated context: 0.04). The SpliceAI-visual bedGraph enabled us to see the existence of an alternative acceptor splice site at position c.225-10 (raw score in mutated context: 0.71). The minigene assay confirmed that the natural splice site is still used predominantly despite its low raw score, and the alternative splice site, which has a higher raw score, is also used, but in a small proportion (Supplemental Figure 2).

Six variants led to both exon skipping and another alteration (2 exonic deletions and 4 intronic retentions) (Supplemental Figure 1E). For the *GCK* c.680-15C>A variant, the natural splice site is not predicted to be significantly altered by SpliceAI (raw score is 1 in the natural context vs. 0.81 in the mutated context). However, this tool predicts the creation of a cryptic splice site at position c.680-13 with a final raw score of 0.58. The minigene assay showed an exon skipping and an alternative transcript with retention of 13 base pairs and no full-length transcript, meaning that, in the mutated context, the alternative splice site is used preferentially despite its lower raw score.

Finally, minigene assays revealed complex and partial splicing patterns for 8 variants (5 missense variants of *GCK* and *HNF4A* and 3 intronic variants located close to the 3′ss of exon 5 of *GCK*) resulting from the activation of distinct splice sites and leading to multiple transcripts (Table 1, Supplemental Table 2, and Supplemental Figure 1F) that were sequenced from each gel band (data not shown). We used SpliceAI-visual bedGraphs to get the raw scores of all predicted splice sites and thus facilitate the analysis of the multiple transcripts generated by these variants. For example, the SpliceAI DS of *GCK* c.1019G>C (last nucleotide of the exon) were as follows: 0.90 (0) for the donor loss site and 0.46 (-17) for the donor gain site, meaning that the natural donor loss site was predicted to be abolished and that a cryptic splice site 17 pb downstream was enhanced. On the SpliceAI-visual representation, an alternative cryptic splice site at position c.1011 was also visible with a DS of 0.11. The raw scores of both the cryptic splice sites are greater than the score of the abolished natural splice site: 0.99 and 0.9 versus 0.03, respectively (Supplemental Figure 2 and Supplemental Table 2). The use of both cryptic splice sites was observed in the minigene assay.

In total, the 33 splicing alterations observed on minigene assays had been predicted by SpliceAI and SPiP except for two variants (*GCK* c.677T>G and *HNF4A* c.225-3C>A) predicted only by SpliceAI, and the *HNF4A* variant c.225-3C>A corresponding to a 3′ physiological splice site was poorly predicted (Figure 4(b)). To be noted, two *GCK* variants (c.580-9T>A and c.1019+20G>A) inducing a splicing alteration had a very low SPiP score, 0.134 and 0.116, respectively, while the median of the SPiP scores for all confirmed defects was 0.938.

However, some discrepancies were observed between the predicted alterations and those observed in the minigene assays (Figure 4(d)). For example, for variants *GCK* c.1019G>A and c.1019G>C, SPiP predicted two alternative splice sites, and only one of them (c.1011) was confirmed by the minigene assay. For 14 other variants, a splicing defect was correctly predicted by SPiP, but the positions of the cryptic or de novo splice sites were not determined. Similarly, some inconsistencies were observed between the expected splice defects predicted by SpliceAI and the minigene results (Figure 4(d)). For example, for the variant *HNF4A* c.426G>A, SpliceAI predicted the diminution of the physiological splice site and the creation of a cryptic splice site at position c.426+8. However, the minigene assay did not show the use of this splice site but the use of another cryptic splice at position c.411, with a SpliceAI raw score lower than the raw score of the physiological and main cryptic splice sites in a variant context (Supplemental Figure 2). This additional cryptic splice site could easily be spotted on the SpliceAI-visual bedGraph, bringing out again the interest of this implement.

In a second step, we compared SpliceAI and SPiP predictions with those of two bioinformatics tools, Pangolin and AbSplice-DNA, recently developed (Table 1 and Supplemental Table 2) [24, 25]. Predictions were concordant with SpliceAI for all variants except two (*GCK* c.863+3A>G and *HNF1A* c.1623G>A not predicted by AbSplice-DNA and Pangolin, respectively). Note that no prediction on deletions, duplications, and delins variants as well as information on the positions of cryptic splice sites was given by AbSplice-DNA.

### 3.3. Reclassification of Spliceogenic Variants

We reviewed the interpretation and classification of spliceogenic variants based on the generic ACMG/AMP criteria [19] combined with the SpliceACORD recommendations for interpretation of RNA functional analysis [27] (Supplemental Table 3).

According to the nature of the RNA splice alteration, we applied the following criteria: (i) PS3 (functional evidence) instead of PP3 (in silico evidence) if the aberrant transcript resulted in an out-of-frame protein, (ii) PM4 instead of PP3 if the abnormal transcript produced an in-frame minigene transcript (except for in-frame exon skipping concerning exons defined as essential for protein function by the monogenic diabetes expert panel and for which PS3 was applied), or (iii) PM1 if the splice site shift affected a well-established functional domain. Furthermore, if a splice defect was demonstrated for a missense *GCK* variant, PP2 criteria (applicable for missense variants of *GCK*) were removed.

For variants with partial splicing defects leading to multiple transcripts, the weakest criterion of pathogenicity was generally considered (i.e., if a variant generated an in-frame alteration and an out-of-frame alteration, the in-frame alteration was considered and the PM4 criterion was used instead of PS3), except if the transcript leading to the use of the strongest criteria was clearly the main splicing product (see, for example, variant GCK c.482AG in Supplemental Table 3). In addition, we decreased the strength of the PS3 (strong) criterion to PS3_M (moderate) if a residual full-length transcript was observed or if the splicing pattern was unclear. When no notable splicing defect was observed with an intronic variant (c.713+10C>T), the BS3 criterion was applied. This criterion was not applied for missense variants, as their putative deleterious effect could not be excluded based on splicing analysis.

Of the 33 variants inducing aberrant splicing, a strong evidence of pathogenicity (PS3) was considered for 24 variants; 15 of them induced out-of-frame splicing alterations (62.5%), 6 led to in-frame alterations (25%), and 3 led to both out-of-frame and in-frame variants (12.5%) (Figure 4(c) and Supplemental Table 3). These 24 variants involved 7 missense, 3 synonymous, and 2 delins variants encompassing the exon-intron junction and 12 noncanonical splice-site intronic variants located as far as 15 and 20 bp from the 3′ss and 5′ss, respectively.

The minigene assays enabled the reclassification of 23/31 (74%) VUS, now considered as likely pathogenic, allowing a definitive molecular diagnosis of monogenic diabetes for 34 families comprising 60 patients with MODY diabetes (Table 1, Figures 4(e) and 4(f), and Supplemental Table 3). Five variants with splicing anomalies (*GCK* c.580-9T>G, c.580-3C>A, c.580-3del, *HNF4A* c.225-3C>A, and c.426G>A p.(Gln142=)) remained classified as VUS as minigene results were unclear showing residual full-length transcript and/or multiple transcripts.

The three other VUS (*GCK* c.356C>G, p.(Ala119Gly); c.484G>A, p.(Gly162Ser); and *HNF1A* c.713+10C>T) showed normal RNA splicing in the minigene assay. The 2 missense GCK variants could still have an impact, impairing the enzyme kinetics rather than RNA missplicing as for the majority of GCK missense variants [5].

## 4. Discussion

In the present study, we analyzed 36 variants involving the three main genes (*GCK*, *HNF1A*, and *HNF4A*) responsible for MODY diabetes and demonstrated by a minigene test that 33 (91.7%) of 36 predicted spliceogenic variants led to an impact on RNA splicing. This provided additional evidence to reclassify 23 VUS as probably pathogenic and thus to have a definitive diagnosis of a genetic subtype of MODY diabetes, which is important for the most appropriate clinical and therapeutic management.

We focused our study on the analysis of variants, both exonic and intronic, located in the vicinity of the acceptor (-50 bp and +20 bp) and donor (-20 bp and +20 bp) splice sites, but not affecting the canonical splice site positions (-1, -2, +1, and +2 bp). The choice of these regions of interest was driven by three objectives: (i) to integrate the minigene assay into the routine genetic diagnosis of monogenic diabetes currently based on targeted sequencing of the coding regions of the diabetes genes and their 20-50 bp flanking intronic regions [3, 4], (ii) to analyze synonymous and missense variants that are potentially spliceogenic, and (iii) to analyze intronic variants located outside of canonical splice sites, routinely classified as VUS in the absence of a functional splicing test.

Variants analyzed by minigene assays were prioritized by a prior in silico analysis based on two prediction tools, SpliceAI and SPiP [21, 23]. We evaluated the predictive accuracy and concordance of splicing tools with the minigene assays. A splicing defect was observed for 97% and 91% of the variants predicted as spliceogenic by SpliceAI and SPiP, respectively. These data validate results previously reported showing the reliability of splicing predictions obtained with SpliceAI compared to other prediction algorithms [11, 28, 29]. The additional use of two recently developed bioinformatics tools, Pangolin and AbSplice-DNA [24, 25], also confirmed the robustness of predictions based on SpliceAI and SPiP. They show the continued improvement of prediction tools and contrast with the poor results reported on the *GCK* gene showing a concordance of only 58% between the in silico analyses based on the Human Splicing Finder algorithm and minigene assays [17].

Nevertheless, experimental data revealed several inconsistencies, particularly for SPiP, on the location of predicted cryptic splice sites. In 20% of the cases, the predicted position of the activated cryptic splice site was inaccurate. Furthermore, in 45% of the cases predicted by SPiP to alter splicing, the location of the cryptic splice site was not given. Discrepancies were also observed to a lesser extent (24%) for SpliceAI predictions. Our study showed that further analysis of SpliceAI data with the SpliceAI-visual tool [22], which allows visualizing the location of all cryptic splice sites with their raw scores in the context of the reference and mutated sequences, facilitates the interpretation of discrepancies between predicted and observed splicing alterations and the analysis of complex splicing profiles leading to multiple transcripts.

Overall, these discordances in the predicted outcomes highlighted the importance of confirming predicted splicing alterations with functional splicing assays.

The minigene assays confirmed an impact on RNA splicing for 33 out of 36 prioritized variants. Among them, 13 (40%) were exonic variants including 9 missense variants and 4 synonymous variants, further supporting that synonymous variants should not systematically be considered as silent variants [30]. The other variants were mostly intronic 17 (53%) affecting either the donor site (60%) or the acceptor site (40%). Finally, two variants were indels located at an exon-intron junction. As previously reported, we observed a different frequency of spliceogenic variants depending on their relative position to the splice site [27, 29]. Variants located at the -1 and +5 bp positions of the splice donor site and at the -3 bp position of the splice acceptor site were, in our cohort, the variants leading to a splicing defect as expected from the consensus motives reported by Cartegni et al. [8] (Figure 2(b)). Nevertheless, our study shows that variants located at more distant positions, at -15 bp of the splice acceptor sites and +20 bp of splice donor sites, could also induce a missplicing event. As our study was limited to a sequence analysis window of -50 bp to +20 bp of the splice acceptor and donor sites, the number of spliceogenic variants was probably underestimated. Further studies are required to determine whether deeper exonic and intronic variants in the three genes analyzed have putative effects on splicing. Of note, a deep intronic variant of GCK (c.483+117T>C) leading to an exon 5 skipping has been reported [17]. Furthermore, other types of exonic variants, such as nonsense and in-frame variants, may also lead to splicing alterations [31–34].

The missplicing events led to a large diversity of splicing alterations consisting of exon skipping, partial exonic deletion/intronic retention, the joined occurrence of exon skipping and another alteration, and complex splicing patterns with multiple transcripts with very close prevalence (15%, 15%, 21%, 18%, and 24%, respectively). This contrasts with variants affecting canonical splice sites, which in about two-thirds of the cases lead to exon skipping. The majority of missplicing events in this study led to out-of-frame transcripts susceptible to nonsense-mediated RNA decay [35].

Reinterpretation of the variants taking into account the minigene results and based on the ACMG criteria and the SpliceACORD consortium recommendations for harmonizing RNA-based diagnostics [27] allowed us to reclassify 23 (74%) of 31 VUS as likely pathogenic. These results underline the contribution of the minigene approach to the reclassification of VUS and the importance of integrating RNA-splicing analysis in the diagnosis process [36]. For five VUS, the minigene assay showed the residual presence of the reference transcript and complex splicing patterns with the detection of several transcripts. With regard to the clinical impact of missplicing defects being difficult to predict in these situations, we have therefore modulated the PS3 criterion to moderate evidence (PS3_M) of missplicing as suggested by the SpliceACORD consortium [27].

Our study has several limitations. We did not consider intronic variants located at canonical splice sites (±1 and ±2 bp) predicted to cause loss of function while some of them may induce in-frame deletions or insertions and not be pathogenic as previously reported [37, 38]. Nevertheless, we are not aware of canonical splicing variants affecting the *GCK*, *HNFA*, and *HNF4A* genes which do not impact splicing [5, 6]. Moreover, our minigene approach is subject to methodological limitations given our aim to incorporate minigene assays into the standard molecular diagnosis for monogenic diabetes. We excluded variants located in the first and last exons requiring specific minigene constructs. When discrepancies are observed between bioinformatics predictions and minigene test results, it may be justified to perform multiple minigene constructs. This is particularly the case for the variant c.356G>C, p.(Ala119Gly), predicted to be spliceogenic by all four algorithms, and for which, an aberrant transcript was detected at a very low expression level with our minigene construct. However, our study showed that such situations remain exceptional, as we were able to reach conclusive results in 92% of the analyzed cases.

## 5. Conclusion

Our study shows the effectiveness of combining several bioinformatics tools and minigene assays to assess the impact of variants on RNA splicing. The current robustness of prediction tools allows a reliable prioritization of spliceogenic variants, thus reducing the number of minigene tests to be performed and allowing the integration of this functional test into routine genetic diagnostic practice. Transcriptomic analysis in patients with clinical suspicion of monogenic diabetes will not be of clinical utility for these three main genes that are not or weakly expressed in blood, highlighting the benefits of the in vitro minigene strategy. Implementing this approach to the genetic diagnostic process will benefit patients with monogenic diabetes for whom the appropriate treatment is determined by the molecular etiology of the disease [1, 2].

## Figures and Tables

**Table 1:** In silico predictions, minigene assay results, and variant classification of 36 variants on *GCK*, *HNF1A*, and *HNF4A* genes.

				Impact on RNA splicing					
				In silico predictions used for variant selection^‡^		Additional in silico predictions^‡^		Minigene assay^§^	
Gene	Variant location^†^		Nucleotide change, predicted protein effect	SpliceAI	SPiP	Pangolin	AbSplice-DNA		Variant classification
*GCK*	Exon 2 (-2)	Donor	c.207A>G, p.(Ser69=)	1	1	1	1	▼E2q10	VUS>LP
*GCK*	Exon 2 (-1)	Donor	c.208G>A, p.(Glu70Lys)	1	1	1	1	▼E2q10	P
*GCK*	Exon 2 (-1)	Donor	c.208G>C, p.(Glu70Gln)	1	1	1	1	▼E2q10	VUS>LP
*GCK*	Intron 2	Donor	c.208+15C>G	1	1	1	1	▼E2q10	VUS>LP
*GCK*	Exon 3 (-8)	Donor	c.356C>G, p.(Ala119Gly)	1	1	1	1	WT	VUS
*GCK*	Exon 4 (-2)	Donor	c.482A>G, p.(Lys161Arg)	1	1	1	1	△**E4**; △E4q24	VUS>LP
*GCK*	Intron 4	Acceptor	c.484-11_484-6del	1	1	1	NA	△E5	VUS>LP
*GCK*	Exon 5 (+1)	Acceptor	c.484G>A, p.(Gly162Ser)	0	1	0	0	WT	VUS
*GCK*	Exon 5 (-1)	Donor	c.579G>T, p.(Gly193=)	1	1	1	1	△E5q2	VUS>LP
*GCK*	Intron 5	Donor	c.579+4del	1	1	1	NA	△E5	VUS>LP
*GCK*	Intron 5	Acceptor	c.580-9T>G	1	1	1	1	▼**E6p27**; ▼E5q109; △E5▼E6p27; WT	VUS
*GCK*	Intron 5	Acceptor	c.580-3C>A	1	1	1	1	▼**E6p27**; ▼E5q109; △E5▼E6p27; WT	VUS
*GCK*	Intron 5	Acceptor	c.580-3del	1	1	1	NA	▼**E6p27**; ▼E5q109; △E5▼E6p27; WT	VUS
*GCK*	Exon 6 (-3)	Donor	c.677T>G, p.(Val226Gly)	1	0	1	1	△E6q16; WT	LP
*GCK*	Exon 6 (-1)	Donor	c.679G>A, p.(Gly227Ser)	1	1	1	1	△E6q16; △E6q4; WT	P
*GCK*	Exon 6 (-1)	Donor	c.679G>C, p.(Gly227Arg)	1	1	1	1	△E6q16; △E6q4; WT	LP
*GCK*	Intron 6	Donor	c.679+5G>A	1	1	1	1	△E6q16; WT	VUS>LP
*GCK*	Intron 6	Acceptor	c.680-15C>A	1	1	1	1	▼E7p13; △E7	VUS>LP
*GCK*	Intron 6	Acceptor	c.680-6C>G	1	1	1	1	▼E7p5	LP>P
*GCK*	Intron 7	Donor	c.863+3A>G	1	1	1	0	△E7q10	VUS>LP
*GCK*	Intron 7	Donor	c.863+5G>A	1	1	1	1	△E7q10	VUS>LP
*GCK*	Exon 8 (-1)	Donor	c.1019G>A, p.(Ser340Asn)	1	1	1	1	△**E8**; △E8q8; ▼E8q17	VUS>LP
*GCK*	Exon 8 (-1)	Donor	c.1019G>C, p.(Ser340Thr)	1	1	1	1	△**E8**; △E8q8; ▼E8q17	VUS>LP
*GCK*	Intron 8	Donor	c.1019+20G>A	1	1	1	1	▼E8q17	VUS>LP
*GCK*	Exon 9–intron 9	Donor	c.1190_1253+11dup	1	1	1	NA	▼E9q75	VUS>LP
*HNF1A*	Intron 2	Donor	c.526+5G>A	1	1	1	1	△E2q32	VUS>LP
*HNF1A*	Intron 3	Donor	c.713+10C>T	0	1	0	0	WT	VUS
*HNF1A*	Intron 4	Donor	c.955+5G>C	1	1	1	1	▼E4q7; △E4	VUS>LP
*HNF1A*	Exon 7 (-1)	Donor	c.1501G>A, p.(Ala501Thr)	1	1	1	1	△E7	VUS>LP
*HNF1A*	Intron 7	Donor	c.1501+4A>G	1	1	1	1	△**E7**; ▼E7q42	VUS>LP
*HNF1A*	Intron 7	Donor	c.1501+5G>C	1	1	1	1	△**E7**; ▼E7q42	VUS>LP
*HNF1A*	Exon 8 (-1)	Donor	c.1623G>A, p.(Gln541=)	1	1	0	1	△E8	VUS>LP
*HNF1A*	Exon 9–intron 9	Donor	c.1742_1768+2delinsACAGGG	1	1	NA	NA	△E9; △E8E9	VUS>LP
*HNF4A*	Intron 2	Acceptor	c.225-3C>A	1	0	1	1	**WT**; ▼E3p10	VUS
*HNF4A*	Exon 4 (-1)	Donor	c.426G>A, p.(Gln142=)	1	1	1	1	△E4q15; △E4; WT	VUS
*HNF4A*	Exon 8 (-1)	Donor	c.1063G>C, p.(Gly355Arg)	1	1	1	1	▼E8q65; △E8q181	VUS>LP

^†^The number in parentheses indicates, for exonic variants, the distance in bp from the splicing site (ss). ^‡^RNA-splicing in silico predictions are detailed in Supplemental Table 2. The number 0 or 1 indicates in silico predictions obtained with each algorithm: “0” if no splicing defect was predicted and “1” in case of a prediction of splicing defect. ^§^Transcripts indicated in bold characters correspond to major transcripts. Variant classification based on a 5-tier system [19]. ▼: intronic retention; △: whole or segmental deletions; E: the affected exon; letters p and q: acceptor and donor splice site shifts, respectively, followed by the number of nucleotides deleted or inserted; WT: the full-length transcript; VUS: variant of unknown significance; LP: likely pathogenic.

## Data Availability

All data relevant to the study are included in the manuscript or uploaded as supplementary information.

## Supplementary Materials

Supplementary 1 Supplemental Figure 1: analysis of the impact on splicing of *GCK*, *HNF1A*, and *HNF4A* variants based on minigene-splicing assay. (A) Variants with no effect on splicing. (B) Variant leading to exon skipping. (C) Variants inducing exonic nucleotide deletion. (D) Variants causing intronic nucleotide retention. (E) Variants leading to exon skipping and another type of missplicing event. (F) Variants causing complex splicing alterations. The pictures show RT-PCR products visualized on agarose gels and annotated as described in Materials and Methods. v: empty pCAS2 vector; WT: wild type; △: nucleotide deletion; ▼: nucleotide insertion.

Supplementary 2 Supplemental Figure 2: visualization of in silico predictions for some variants tested by minigene assay. For each variant is presented the results of SPiP (interpretation, interconfident value, main SPiP score, and predicted alternative site if applicable), SpliceAI (500 pb window), and SpliceAI-visual accessed via the MobiDetails website (https://mobidetails.iurc.montp.inserm.fr/MD/).

Supplementary 3 Supplemental Table 1: list of primers and pCAS2-*GCK*, pCAS2-*HNF1A*, and pCAS2-*HNF4A* minigene constructs used in this study.

Supplementary 4 Supplemental Table 2: bioinformatics predictions of splicing alterations for the 36 variants selected for minigene assays on *GCK*, *HNF1A*, and *HNF4A* and comparison with experimental data.

Supplementary 5 Supplemental Table 3: classification of variants tested in minigene assays.
