# Supplementary figures and images for: Evaluation in Monogenic Diabetes of the Impact of GCK, HNF1A, and HNF4A Variants on Splicing through the Combined Use of In Silico Tools and Minigene Assays
Source: Hum Mutat. 2023 Aug 31;2023:6661013. doi: 10.1155/2023/6661013 (PMC11919142; doi:10.1155/2023/6661013)

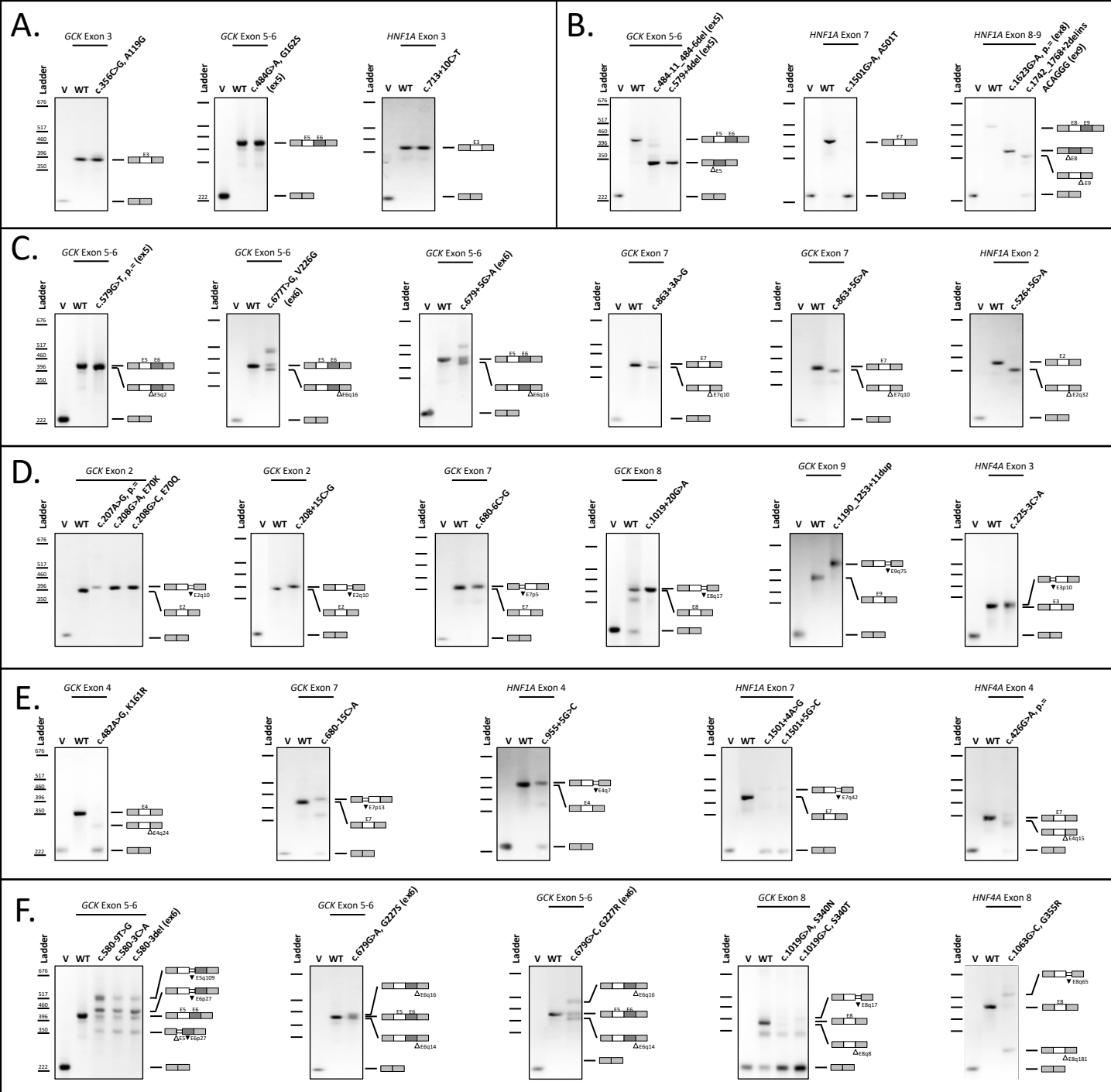

Supplement: Supplementary 1 — Supplemental Figure 1: analysis of the impact on splicing of GCK, HNF1A, and HNF4A variants based on minigene-splicing assay. (A) Variants with no effect on splicing. (B) Variant leading to exon skipping. (C) Variants inducing exonic nucleotide deletion. (D) Variants causing intronic nucleotide retention. (E) Variants leading to exon skipping and another type of missplicing event. (F) Variants causing complex splicing alterations. The pictures show RT-PCR products visualized on agarose gels and annotated as described in Materials and Methods. v: empty pCAS2 vector; WT: wild type; △: nucleotide deletion; ▼: nucleotide insertion. [file 6661013.f1.pdf]
