# Supplementary material for: Evaluation in Monogenic Diabetes of the Impact of GCK, HNF1A, and HNF4A Variants on Splicing through the Combined Use of In Silico Tools and Minigene Assays
Source: Hum Mutat. 2023 Aug 31;2023:6661013. doi: 10.1155/2023/6661013 (PMC11919142; doi:10.1155/2023/6661013)
Supplement: Supplementary 2 — Supplemental Figure 2: visualization of in silico predictions for some variants tested by minigene assay. For each variant is presented the results of SPiP (interpretation, interconfident value, main SPiP score, and predicted alternative site if applicable), SpliceAI (500 pb window), and SpliceAI-visual accessed via the MobiDetails website (https://mobidetails.iurc.montp.inserm.fr/MD/). [file 6661013.f2.pdf]

Supplemental figure S2
Visualization of *in silico* predictions for some variants tested by minigene assay

GCK:
- c.356C>G: no splicing defect (SPiP and SpliceAI false positive)
- c.482A>G: a cryptic splice site with high SpliceAI row score (RS) not used in minigene assay
- c.484G>A: no splicing defect (SPiP false positive)
- c.580-9T>C, c.580-3C>A and c.580-3del: complex alteration
- c.677T>G: SPiP false negative
- c.679G>A and c.679G>C: complex alteration
- c.680-15C>A: use of a cryptic splice site with lower RS
- c.863+3A>G and c.863+5G>A: importance of SpliceAI Visual representation
- c.1019G>A and c.1019G>C: complex alteration

HNF1A:
- c.713+10C>T: no splicing defect (SPiP false positive)
- c.1501G>A: exon skipping despite physiological splice site predited to be moderatly affected
- c.1623G>A: importance of SpliceAI Visual representation

HNF4A:
- c.225-3C>A: SPiP false negative
- c.426G>A: importance of SpliceAI Visual representation
- c.1063G>C: complex alteration

GCK c.356C>G, p.(Ala119Gly) (ex3)

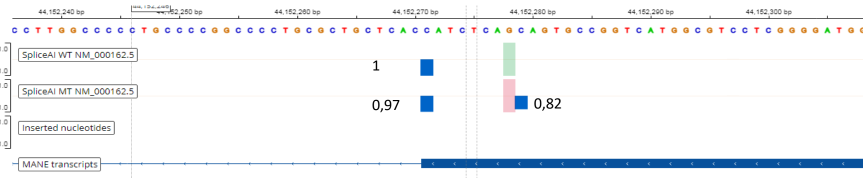

spliceAI lookup (500) AG:	0.00 (101)
spliceAI lookup (500) AL:	0.00 (85)
spliceAI lookup (500) DG:	0.82 (1)
spliceAI lookup (500) DL:	0.03 (-7)

SPiP results and predictions:

Features	Values
Interpretation	+ Creation of a new splice site + Alteration of an exonic splicing regulatory element
Risk	69.33 % [61.29 % - 76.59 %]

SPiP Score	0,602
posCryptMut	44152278 (c.355)
probaCryptMut	0,3138341
posSSPhysio	44152270 (c.363)
probaSSPhysioMut	0,3473684

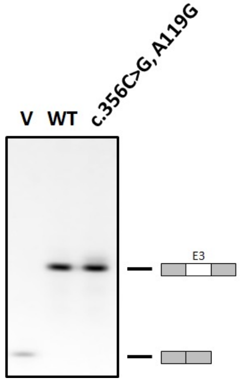

Conclusion/interpretation:

All algorithms predicted a splicing alteration (with the creation of an alternative splice site at position c.355; with a SpliceAI raw score lower than the score of the natural splice site though).

We indeed observed the use of this cryptic splice site (which induces the alteration ΔE3q8) but at a very low proportion and considered it insignificant. As a result, we concluded that this variant induced no splicing defect.

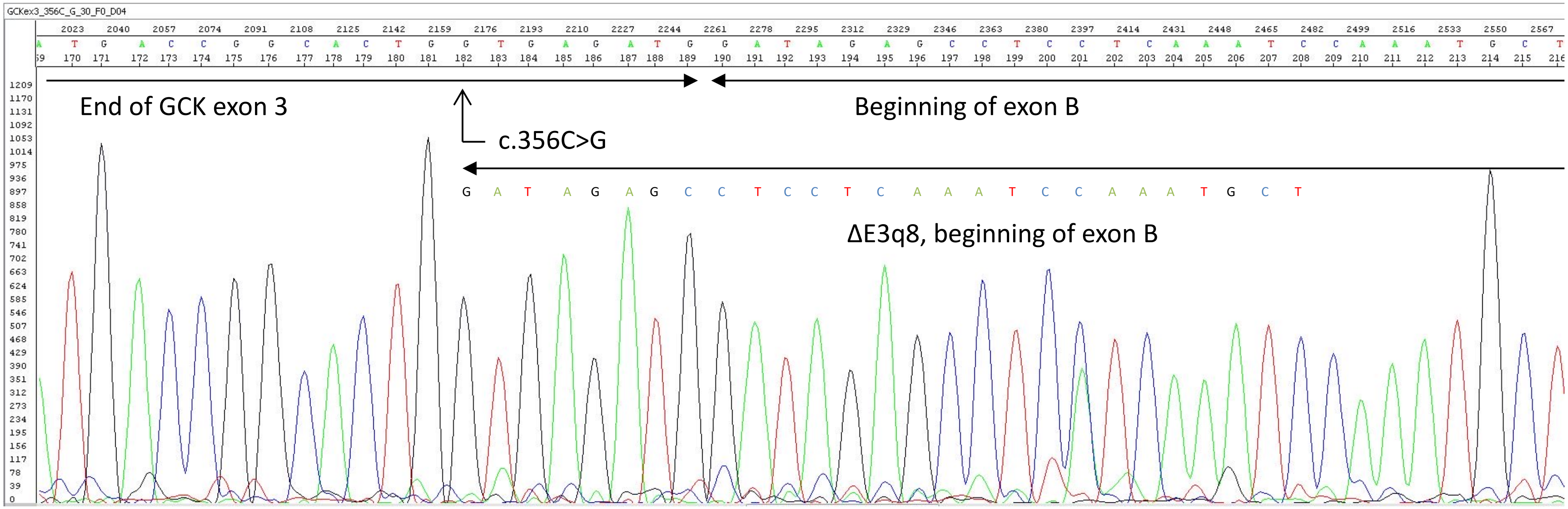

GCK c.484G>A, p.(Gly162Ser) (ex5)

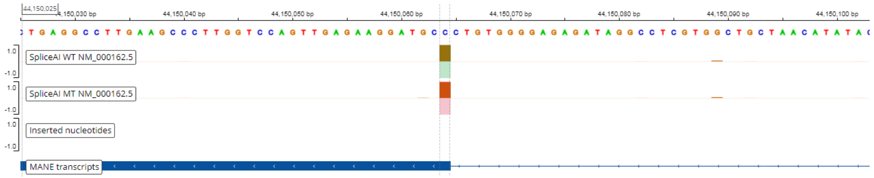

spliceAI lookup (500) AG:	0.01 (-2)
spliceAI lookup (500) AL:	0.02 (0)
spliceAI lookup (500) DG:	0.00 (70)
spliceAI lookup (500) DL:	0.00 (-95)

SPiP results and predictions:

Features	Values
Interpretation	Alteration of the consensus splice site
Risk	35.81 % [28.11 % - 44.1 %]

SPiP Score	0,304
posCryptMut	44150062 (c.486)
probaCryptMut	0,0003394945
posSSPhysio	44150064 (c.484)
probaSSPhysioMut	0,42976

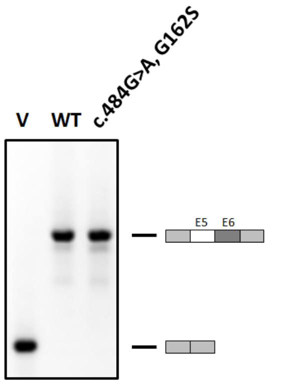

Conclusion/interpretation:

SPiP was the only algorithm (out of 4) to predict a splicing alteration. Minigene assay showed no splicing defect (SPiP false positive).

GCK c.677T>G, p.(Val226Gly) (ex6)

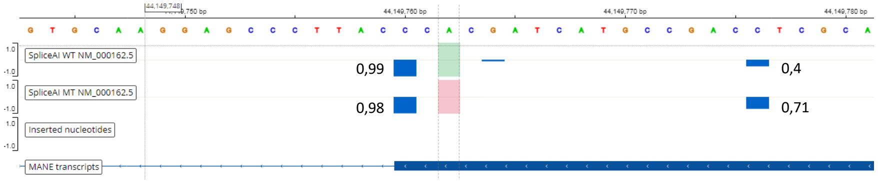

spliceAI lookup (500) AG:	0.00 (-62)
spliceAI lookup (500) AL:	0.00 (124)
spliceAI lookup (500) DG:	0.31 (14)
spliceAI lookup (500) DL:	0.00 (2)

SPiP results and predictions:

Features	Values
Interpretation	No effect on splicing
Risk	09.3 % [02.59 % - 22.14 %]

SPiP score : 0,082

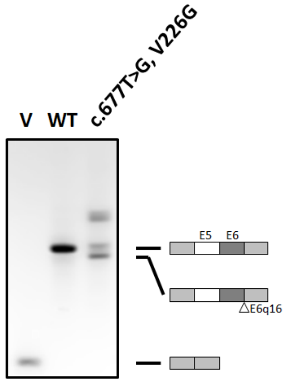

Conclusion/interpretation:

SpliceAI predicted the increase of a cryptic splice site at position c.663, but with a raw score lower than the score of the natural splice site. The use of this site was indeed seen in the minigene assay.

GCK c.580-9T>G (ex6)

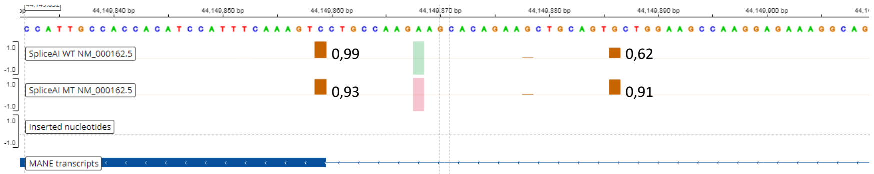

spliceAI lookup (500) AG:	0.29 (18)
spliceAI lookup (500) AL:	0.06 (-9)
spliceAI lookup (500) DG:	0.01 (-92)
spliceAI lookup (500) DL:	0.00 (101)

SPiP results and predictions:

Features	Values
Interpretation	Alteration of the consensus splice site
Risk	23.61 % [16.94 % - 31.4 %]

SPiP score : 0,134

GCK c.580-3C>A (ex6)

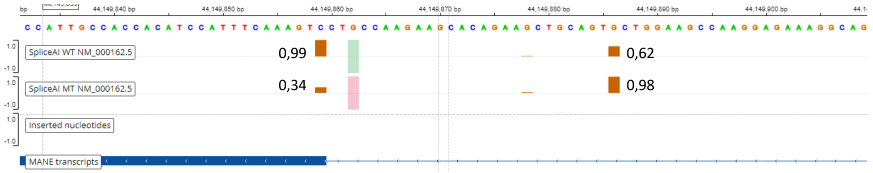

spliceAI lookup (500) AG:	0.37 (24)
spliceAI lookup (500) AL:	0.65 (-3)
spliceAI lookup (500) DG:	0.03 (-86)
spliceAI lookup (500) DL:	0.00 (107)

SPiP results and predictions:

Features	Values
Interpretation	Alteration of the consensus splice site
Risk	98.41 % [91.47 % - 99.96 %]

SPiP score : 0,972

GCK c.580-3del (ex6)

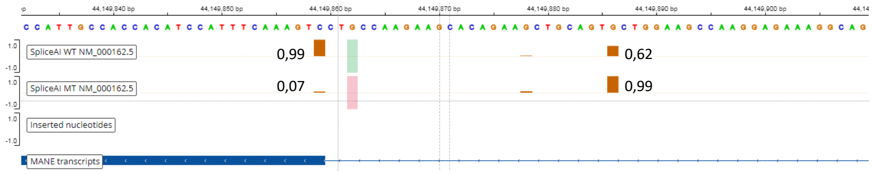

spliceAI lookup (500) AG:	0.38 (25)
spliceAI lookup (500) AL:	0.92 (-2)
spliceAI lookup (500) DG:	0.00 (125)
spliceAI lookup (500) DL:	0.00 (-85)

SPiP results and predictions:

Features	Values
Interpretation	Alteration of the consensus splice site
Risk	98.41 % [91.47 % - 99.96 %]

SPiP score : 0,988

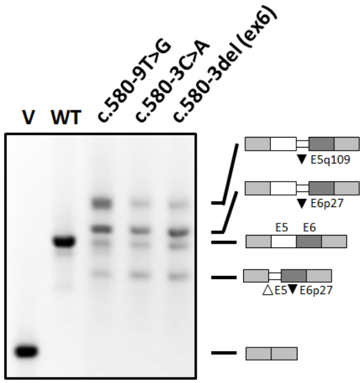

Conclusion/interpretation:

Minigene assay showed several transcripts:

- Total retention of intron 5
- Retention of the last 27 nucleotides of intron 5
- Residual full-length transcript
- Loss of exon 5 and retention of the last 27 nucleotides of intron 5

Which is a more complexe situation than expected,

GCK c.679G>A, p.(Gly227Ser) (ex6)

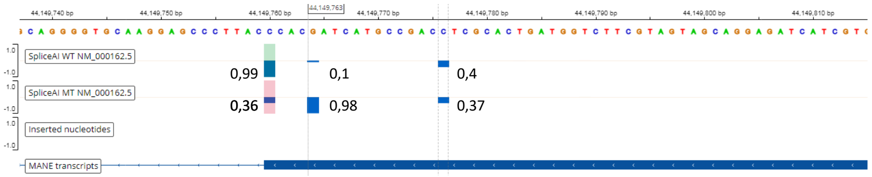

spliceAI lookup (500) AG:	0.00 (126)
spliceAI lookup (500) AL:	0.00 (-120)
spliceAI lookup (500) DG:	0.88 (4)
spliceAI lookup (500) DL:	0.63 (0)

SPiP results and predictions:

Features	Values
Interpretation	+ Alteration of the consensus splice site + Creation of a new splice site
Risk	85.91 % [79.27 % - 91.06 %]
0.764	Main SPiP score
44149763 = c.675	Position of the strongest cryptic site reinforced by the mutation
Don	Splice site type of the cryptic site
0.4110537	Cryptic score

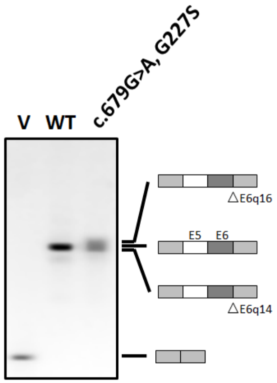

GCK c.679G>C, p.(Gly227Arg) (ex6)

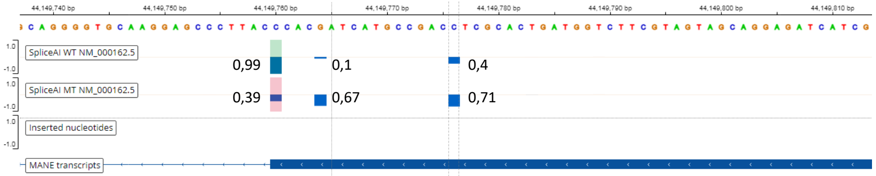

spliceAI lookup (500) AG:	0.01 (118)
spliceAI lookup (500) AL:	0.00 (126)
spliceAI lookup (500) DG:	0.57 (4)
spliceAI lookup (500) DL:	0.60 (0)

SPiP results and predictions:

Features	Values
Interpretation	+ Alteration of the consensus splice site + Creation of a new splice site
Risk	96.71 % [92.49 % - 98.92 %]
0.768	Main SPiP score
44149763 = c.675	Position of the strongest cryptic site reinforced by the mutation
Don	Splice site type of the cryptic site
0.09615555	Cryptic score

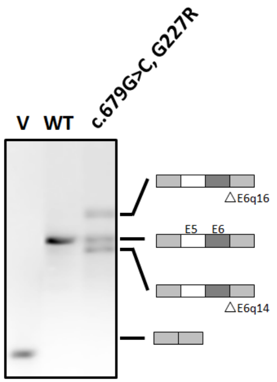

Conclusion/interpretation for the two variants:

SpliceAI predicted the decrease of the natural splice site and the increase of a cryptic splice site at position c.675. SpliceAI Visual enabled us to see that another cryptic splice site at position c.663 could be used. Minigene assay showed the use of both cryptic splice sites (ΔE6q4 and ΔE6q14 respectively).

GCK c.482A>G, p.(Lys161Arg) (ex5)

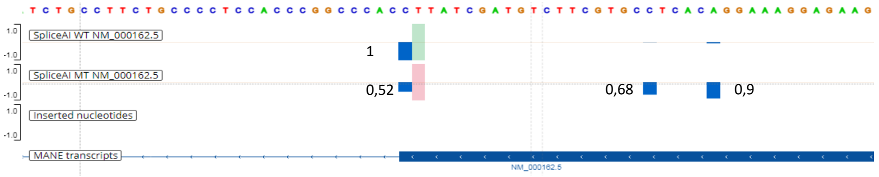

spliceAI lookup (500) AG:	0.01 (145)
spliceAI lookup (500) AL:	0.01 (118)
spliceAI lookup (500) DG:	0.83 (23)
spliceAI lookup (500) DL:	0.48 (-1)

SPiP results and predictions:

Features	Values
Interpretation	+ Alteration of the consensus splice site + Alteration of an exonic splicing regulatory element
Risk	100 % [97.32 % - 100 %]

SPiP score : 0,858

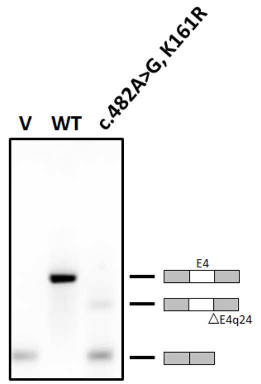

Conclusion/interpretation:

SpliceAI predicted a decrease of the natural splice site and the increase of a site at position c.459. We indeed saw the use of this cryptic site in the minigene assay.

However, SpliceAI Visual showed another cryptic splice site at position c.464 with an higher raw score than the score of the natural splice site. This cryptic splice site does not seem to be used.

GCK c.680-15C>A (ex7)

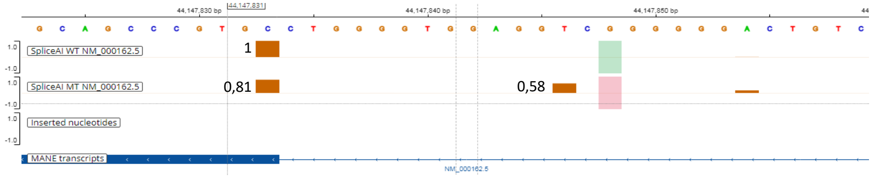

spliceAI lookup (500) AG:	0.58 (-2)
spliceAI lookup (500) AL:	0.18 (-15)
spliceAI lookup (500) DG:	0.00 (-477)
spliceAI lookup (500) DL:	0.01 (-188)

SPiP results and predictions:

Features	Values
Interpretation	Alteration of the polypyrimidine tract (-20 to -18)
Risk	98.41 % [91.47 % - 99.96 %]

SPiP score : 0,94

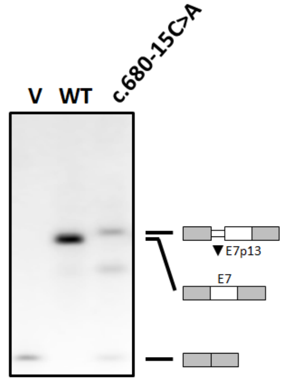

Conclusion/interpretation:

SpliceAI predicted the creation of a cryptic splice site at position c.680-13, but with a raw score lower than the score of the natural splice site. The use of this site was indeed seen in the minigene assay.

GCK c.863+3A>G (ex7)

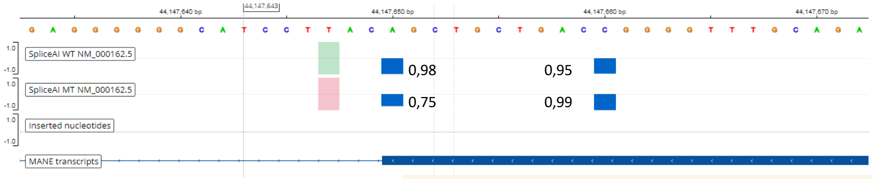

spliceAI lookup (500) AG: 0.00 (-215)
spliceAI lookup (500) AL: 0.00 (-234)
spliceAI lookup (500) DG: 0.08 (-130)
spliceAI lookup (500) DL: 0.23 (3)

SPiP results and predictions:

Features	Values
Interpretation	Alteration of the consensus splice site
Risk	100 % [97.32 % - 100 %]

SPiP score : 0,854

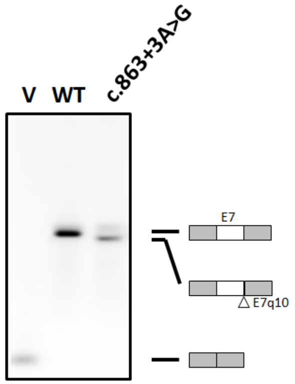

GCK c.863+5G>A (ex7)

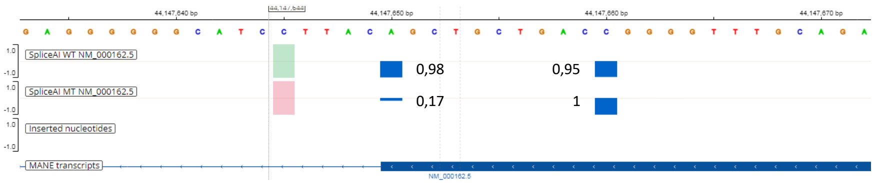

spliceAI lookup (500) AG: 0.00 (-208)
spliceAI lookup (500) AL: 0.02 (-232)
spliceAI lookup (500) DG: 0.09 (53)
spliceAI lookup (500) DL: 0.81 (5)

SPiP results and predictions:

Features	Values
Interpretation	Alteration of the consensus splice site
Risk	98.41 % [91.47 % - 99.96 %]

SPiP score : 0,998

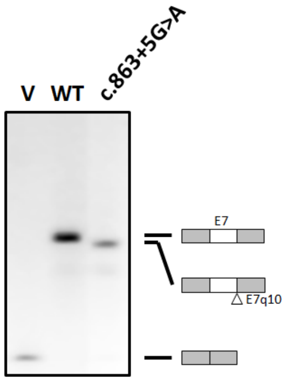

Conclusion/interpretation for the two variants:

SpliceAI predicted only the decrease of the natural splice site. SpliceAI Visual enabled us to see that a cryptic splice site is located at position c.853. The use of this splice site was indeed seen in the minigene assay.

GCK c.1019G>A, p.(Ser340Asn) (ex8)

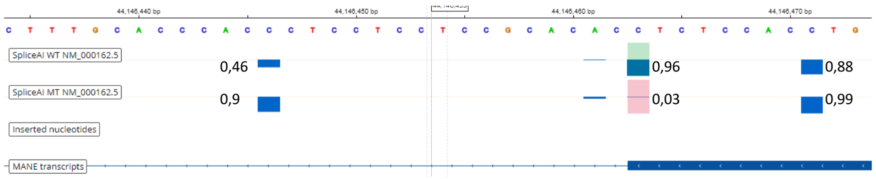

SPiP results and predictions:

Features	Values
Interpretation	Alteration of the consensus splice site
Risk	98.41 % [91.47 % - 99.96 %]
0.926	Main SPiP score
44146460 = c.1019+2	Position of the strongest cryptic site reinforced by the mutation
Don	Splice site type of the cryptic site
0.001333257	Cryptic score
44146470 = c.1011	Position of the strongest cryptic site in wild-type context
0.01884596	Score of the strongest cryptic site in wild-type context

spliceAI lookup (500) AG:	0.01 (95)
spliceAI lookup (500) AL:	0.00 (-482)
spliceAI lookup (500) DG:	0.44 (-17)
spliceAI lookup (500) DL:	0.94 (0)

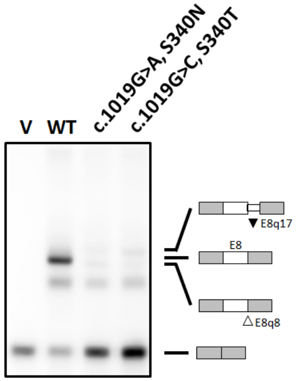

GCK c.1019G>C, p.(Ser340Thr) (ex8)

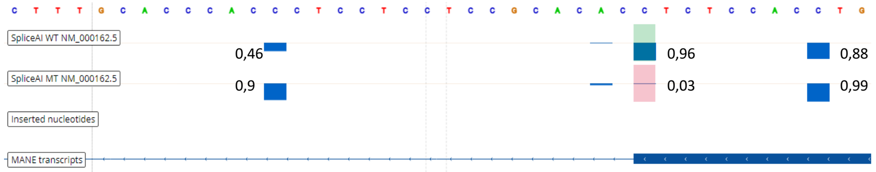

SPiP results and predictions:

Features	Values
Interpretation	Alteration of the consensus splice site
Risk	98.41 % [91.47 % - 99.96 %]
0.928	Main SPiP score
44146460 = c.1019+2	Position of the strongest cryptic site reinforced by the mutation
Don	Splice site type of the cryptic site
0.003168868	Cryptic score
44146470 = c.1011	Position of the strongest cryptic site in wild-type context
0.01884596	Score of the strongest cryptic site in wild-type context

spliceAI lookup (500) AG:	0.01 (95)
spliceAI lookup (500) AL:	0.00 (-482)
spliceAI lookup (500) DG:	0.45 (-17)
spliceAI lookup (500) DL:	0.93 (0)

Conclusion/interpretation for the two variants:

SpliceAI predicted the decrease of the natural splice site and the increase of a cryptic splice site at position c.1019+17. SpliceAI Visual enabled us to see another cryptic splice site at position c.1011, with a lowest DS but a higher RS. The use of both these sites was seen in minigene assay (inducing ▼E8p17 and ΔE8q8 respectively).

HNF1A c.713+10C>T (ex3)

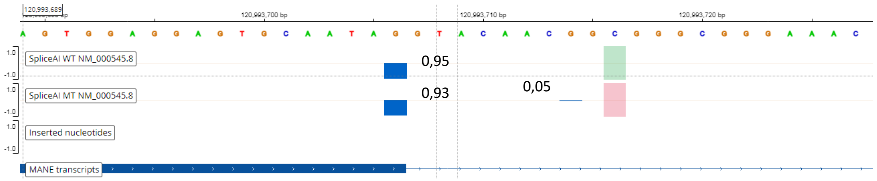

spliceAI lookup (500) AG:	0.00 (485)
spliceAI lookup (500) AL:	0.00 (-196)
spliceAI lookup (500) DG:	0.05 (-2)
spliceAI lookup (500) DL:	0.02 (-10)

SPiP results and predictions:

Features	Values
Interpretation	Creation of a new splice site
Risk	47.89 % [39.44 % - 56.42 %]

SPiP Score	0,43
posCryptMut	120993714 (c.713+8)
probaCryptMut	0,060468411
posSSPhysio	120993706 (c.713)
probaSSPhysioMut	0,006138913

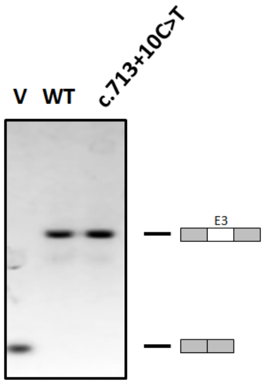

Conclusion/interpretation:

SPiP predicted a splicing alteration *via* the creation of a new splice site. However, the probability of the cryptic splice site (c.713+8) was lower than the probability of the physiological splice site (c.713). Thus, it is consistent with the fact that minigene assay dit not show any splicing defect.

HNF1A c.1501G>A, p.(Ala501Thr) (ex7)

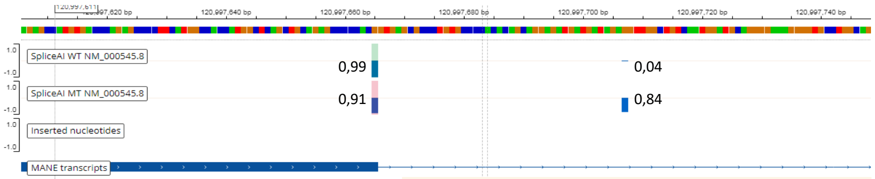

spliceAI lookup (500) AG:	0.00 (25)
spliceAI lookup (500) AL:	0.00 (-174)
spliceAI lookup (500) DG:	0.80 (42)
spliceAI lookup (500) DL:	0.08 (0)

SPiP results and predictions:

Features	Values
Interpretation	+ Alteration of the consensus splice site + Alteration of an exonic splicing regulatory element
Risk	98.41 % [91.47 % - 99.96 %]

SPiP score : 0,954

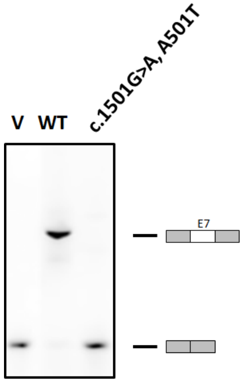

Conclusion/interpretation:

SpliceAI predicted the increase of a cryptic splice site at position c.1501+42. However, this site has a lower RS than the natural splice site. In the end, the minigene assay showed an exon skipping, even though the natural splice site is scarcely affected.

HNF1A c.1623G>A, p.= (ex8)

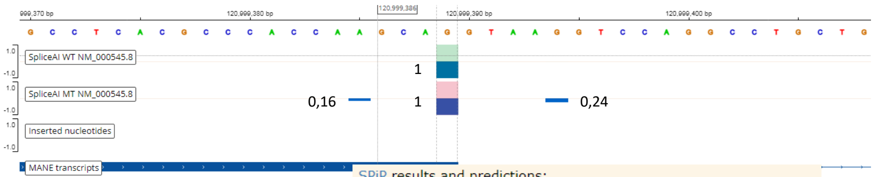

spliceAI DL:	0.01 (0)
spliceAI lookup (500) AG:	0.00 (6)
spliceAI lookup (500) AL:	0.00 (73)
spliceAI lookup (500) DG:	0.24 (5)
spliceAI lookup (500) DL:	0.01 (0)

SPiP results and predictions:

Features	Values
Interpretation	Alteration of the consensus splice site
Risk	54 % [45.68 % - 62.16 %]
0.502	Main SPiP score
120999394 = c.1623+5	Position of the strongest cryptic site reinforced by the mutation
Don	Splice site type of the cryptic site
0.0001958564	Cryptic score

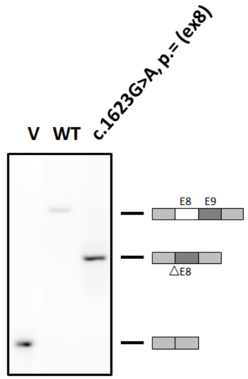

Conclusion/interpretation:

SpliceAI predicted the increase of a cryptic splice site at position c.1623+5. However, this site has a lower RS than the natural splice site. In the end, the minigene assay showed an exon skipping, even though the natural splice site is scarcely affected.

HNF4A c.225-3C>A (ex3)

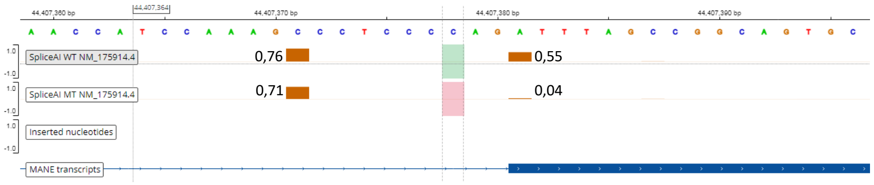

spliceAI lookup (500) AG:	0.02 (-187)
spliceAI lookup (500) AL:	0.51 (3)
spliceAI lookup (500) DG:	0.00 (-7)
spliceAI lookup (500) DL:	0.00 (97)

SPiP results and predictions:

Features	Values
Interpretation	No effect on splicing
Risk	01.85 % [00.38 % - 05.32 %]

SPiP score : 0,018

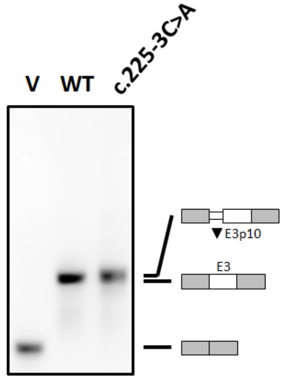

Conclusion/interpretation:

SPiP dit not correctly predict this alteration (false-negative).

HNF4A c.426G>A, p.(Gln142=) (ex4)

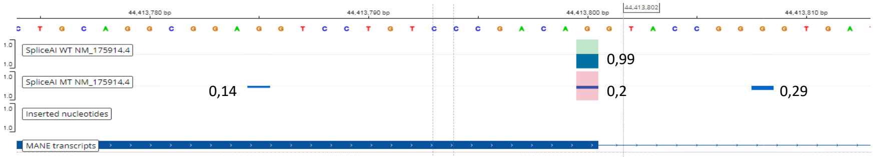

spliceAI lookup (500) AG:	0.00 (8)
spliceAI lookup (500) AL:	0.00 (-106)
spliceAI lookup (500) DG:	0.29 (8)
spliceAI lookup (500) DL:	0.79 (0)

SPiP results and predictions:

Features	Values
Interpretation	Alteration of the consensus splice site
Risk	98.41 % [91.47 % - 99.96 %]

SPiP score : 1

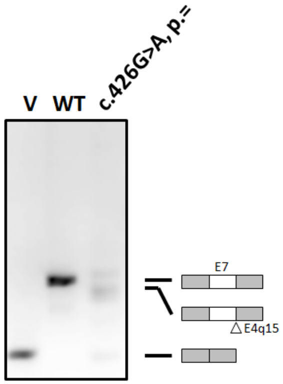

Conclusion/interpretation:

SpliceAI predicted the decrease of the natural splice site and the creation of a cryptic splice site at position c.426+8. SpliceAI Visual showed us that an other cryptic splice site (with a lower RS) is present at position c.411. It is this cryptic splice site that was shown to be used in the minigene assay, inducing the ΔE4q15 alteration.

HNF4A c.1063G>C, p.(Gly355Arg) (ex8)

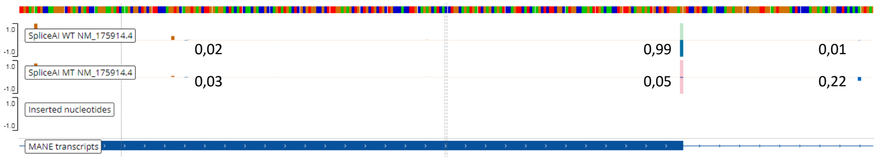

spliceAI lookup (500) AG:	0.00 (-15)
spliceAI lookup (500) AL:	0.00 (-236)
spliceAI lookup (500) DG:	0.21 (65)
spliceAI lookup (500) DL:	0.94 (0)

SPiP results and predictions:

Features	Values
Interpretation	Alteration of the consensus splice site
Risk	98.41 % [91.47 % - 99.96 %]
0.99	Main SPiP score
44424258 = c.1063+4	Position of the strongest cryptic site reinforced by the mutation
Don	Splice site type of the cryptic site
0.000006151665	Cryptic score

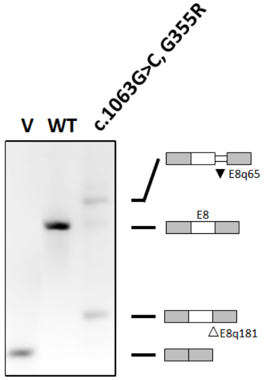

Conclusion/interpretation:

SpliceAI predicted the decrease of the natural splice site and the creation of a cryptic splice site at position c.1063+65. SpliceAI Visual showed us that an other cryptic splice site (with a very low RS) is present at position c.882. Both these cryptic splice sites were shown to be used in the minigene assay, inducing the ▼E8q65 and the ΔE8q181 alterations respectively.
