# Supplementary material for: Evaluation in Monogenic Diabetes of the Impact of GCK, HNF1A, and HNF4A Variants on Splicing through the Combined Use of In Silico Tools and Minigene Assays
Source: Hum Mutat. 2023 Aug 31;2023:6661013. doi: 10.1155/2023/6661013 (PMC11919142; doi:10.1155/2023/6661013)
Supplement: Supplementary 3 — Supplemental Table 1: list of primers and pCAS2-GCK, pCAS2-HNF1A, and pCAS2-HNF4A minigene constructs used in this study. [file 6661013.f3.pdf]

Supplementary Table S1. List of primers and pCAS2-*GCK* , -*HNF1A* and -*HNF4A* minigene constructs used in this study.

Purpose	Forward (F) and Reverse (R) primers		pCAS2 minigenes		Variants tested in minigene splicing assay			
	Name	Sequence	Exons	Coordinates (5' or 3')	Location	ucleotide nomenclatu	Size of PCR product	Size of WT minigene product
PCR (cloning / minigene preparation)	GCK_Ex2F_SPL	CAACACGCTGTGGGCTGCAT	2	c.46-261	exon 2 (-2)<br>exon 2 (-1)<br>exon 2 (-1)<br>intron 2	c.207A>G<br>c.208G>A<br>c.208G>C<br>c.208+15C>G	595	398
	GCK_Ex2R_SPL	GCCGGACCAGAGGAGCCAAG		c.208+171				
	GCK_Ex3F_SPL	CTTTTCCCTGGTTGACCTTTGACCC	3	c.209-205	exon 3 (-8)	c.356C>G	598	390
	GCK_Ex3R_SPL	CACTAACTCTTCCTCAGCCCGTTCC		c.363+238				
	GCK_Ex4F_SPL	ACTGTCAGCAGGTCTCTGGCATG	4	c.364-211	exon 4 (-2)	c.482A>G	546	355
	GCK_Ex4R_SPL	GGTGATCATAGCTGGTGCCTCACAA		c.483+215				
	GCK_Ex5F_SPL	TCTTCAAGGAGAATCGTTCCCA	5-6	c.484-176	intron 4<br>exon 5 (+1)<br>exon 5 (-1)<br>intron 5<br>intron 5<br>intron 5<br>intron 5<br>exon 6 (-3)<br>exon 6 (-1)<br>exon 6 (-1)<br>intron 6	c.484-11_484-6del<br>c.484G>A<br>c.579G>T<br>c.579+4del<br>c.580-9T>G<br>c.580-3C>A<br>c.580-3del<br>c.677T>G<br>c.679G>A<br>c.679G>C<br>c.679+5G>A	696	431
	GCK_Ex6R_SPL	TGCAGGAATGTGGCATCATGGC		c.679+215				
	GCK_Ex7F_SPL	TTGCCTGTTAGGAAAGAGGACAGCC	7	c.680-213	intron 6<br>intron 6<br>intron 7<br>intron 7	c.680-15C>A<br>c.680-6C>G<br>c.863+3A>G<br>c.863+5G>A	579	419
	GCK_Ex7R_SPL	CATCTCTCACAGGGGCAGGTCATG		c.863+182				
	GCK_Ex8F_SPL	GCTCTGGCTCATTAACGAGGGAAAGA	8	c.864-176	exon 8 (-1)<br>exon 8 (-1)<br>intron 8	c.1019G>A<br>c.1019G>C<br>c.1019+20G>A	786	391
	GCK_Ex8R_SPL	GCCTTGGGGTTGTGAGTGATGT		c.1020-279				
	GCK_Ex9F_SPL	TAGAGGGGGCAGTACTAACCAGTCC	9	c.1020-230	intron 9	c.1190_1253+11dup	647	469
	GCK_Ex9R_SPL	CTCCCACCTCATCCTCCACATTCAT		c.1253-34				
	HNF1A_Ex2F_SPL	CCTACCATCCATCCACCAGT	2	c.327-481	intron 2	c.526+5G>A	866	435
	HNF1A_Ex2R_SPL	GTCTACTCCCGTCCCACAGA		c.526+185				
	HNF1A_Ex3F_SPL	CTTGGGTTGGTAGGAAAGCA	3	c.527-245	intron 3	c.713+10C>T	830	422
	HNF1A_Ex3R_SPL	ATGAAGGGGAGGGTTCTGAG		c.714-60				
	HNF1A_ex4F_SPL	TGTCCAGTTGCCGAGAACTC	4	c.713+91	intron 4	c.955+5G>C	844	477
	HNF1A_ex4R_SPL	GGAATGGGGTTAATTGTGGTGG		c.955+235				
	HNF1A_Ex7F_SPL	ATGACTTGCCAGAGCCACTT	7	c.1310-180	exon 7 (-1)<br>intron 7<br>intron 7	c.1501G>A<br>c.1501+4A>G<br>c.1501+5G>C	604	427
	HNF1A_Ex7R_SPL	TACACACCCAGACACGCACT		c.1501+232				
	HNF1A_Ex8F_SPL	AGCTGAGCAGTTCCCTGTAATG	8-9	c.1502-202	exon 8 (-1)<br>exon 9	c.1623G>A<br>c.1742_1768+2delins	798	502
	HNF1A_Ex9R_SPL	CAGTCCAGTCCCTGAGATGTTC		c.1768+236				
	HNF4A_Ex3F_SPL	ACTCAGCTCTAACACCAACCAG	3	c.225-268	intron 2	c.225-3C>A	577	330
	HNF4A_Ex3R_SPL	AGAGTGGGAGCTTATCTCTCTGA		c.319+214				
	HNF4A_Ex4F_SPL	GCTGATGGGTGGATCACTCTTA	4	c.320-234	exon 4 (-1)	c.426G>A	600	342
	HNF4A_Ex4R_SPL	CAGTGAAGGTGAAGACTCTGCT		c.426+259				
	HNF4A_Ex8F_SPL	TCTGCCTGTGTCTAGGAAATCA	8	c.827-186	exon 8 (-1)	c.1063G>C	600	472
	HNF4A_Ex8R_SPL	GACCATTGCCTTGTTCCCAT		c.1063+177				
sequencing of minigene inserts	pCAS-Seq-F	GGGGTCAATAGCAGTGAGAGG						
	pCAS-Seq-R	GCTCCATTTCACAGGTAGAGA						
RT-PCR and sequencing of RT-PCR products	pCAS-KOI-F	TGACGTCGCCGCCCATCAC						
	pCAS-2R	ATTGGTTGTTGAGTTGGTTGTC						

the forward primers used for PCR all begin with: AAGAAGTGCAGGATC; and all reverse primers begin with: TCAAAACAAGACGCG (complementary sequence to the pCAS2 plasmid necessary for the insertion of the PCR product in the linearized vector via InFusion cloning kit.)

For the exons 5 and 6 of *GCK* and exons 8 and 9 of *HNF1A* , those short and closely located exons were cloned together in the vector.
