# Supplementary material for: Evaluation in Monogenic Diabetes of the Impact of GCK, HNF1A, and HNF4A Variants on Splicing through the Combined Use of In Silico Tools and Minigene Assays
Source: Hum Mutat. 2023 Aug 31;2023:6661013. doi: 10.1155/2023/6661013 (PMC11919142; doi:10.1155/2023/6661013)
Supplement: Supplementary 4 — Supplemental Table 2: bioinformatics predictions of splicing alterations for the 36 variants selected for minigene assays on GCK, HNF1A, and HNF4A and comparison with experimental data. [file 6661013.f4.pdf]

Supplementary Table S1. List of primers and pCAS2-*GCK* , -*HNF1A* and -*HNF4A* minigene constructs used in this study.

Purpose	Forward (F) and Reverse (R) primers		pCAS2 minigenes		Variants tested in minigene splicing assay			
	Name	Sequence	Exons	Coordinates (5' or 3')	Location	ucleotide nomenclatu	Size of PCR product	Size of WT minigene product
PCR (cloning / minigene preparation)	GCK_Ex2F_SPL	CAACACGCTGTGGGCTGCAT	2	c.46-261	exon 2 (-2)<br>exon 2 (-1)<br>exon 2 (-1)<br>intron 2	c.207A>G<br>c.208G>A<br>c.208G>C<br>c.208+15C>G	595	398
	GCK_Ex2R_SPL	GCCGGACCAGAGGAGCCAAG		c.208+171				
	GCK_Ex3F_SPL	CTTTTCCCTGGTTGACCTTTGACCC	3	c.209-205	exon 3 (-8)	c.356C>G	598	390
	GCK_Ex3R_SPL	CACTAACTCTTCCTCAGCCCGTTCC		c.363+238				
	GCK_Ex4F_SPL	ACTGTCAGCAGGTCTCTGGCATG	4	c.364-211	exon 4 (-2)	c.482A>G	546	355
	GCK_Ex4R_SPL	GGTGATCATAGCTGGTGCCTCACAA		c.483+215				
	GCK_Ex5F_SPL	TCTTCAAGGAGAATCGTTCCCA	5-6	c.484-176	intron 4<br>exon 5 (+1)<br>exon 5 (-1)<br>intron 5<br>intron 5<br>intron 5<br>intron 5<br>exon 6 (-3)<br>exon 6 (-1)<br>exon 6 (-1)<br>intron 6	c.484-11_484-6del<br>c.484G>A<br>c.579G>T<br>c.579+4del<br>c.580-9T>G<br>c.580-3C>A<br>c.580-3del<br>c.677T>G<br>c.679G>A<br>c.679G>C<br>c.679+5G>A	696	431
	GCK_Ex6R_SPL	TGCAGGAATGTGGCATCATGGC		c.679+215				
	GCK_Ex7F_SPL	TTGCCTGTTAGGAAAGAGGACAGCC	7	c.680-213	intron 6<br>intron 6<br>intron 7<br>intron 7	c.680-15C>A<br>c.680-6C>G<br>c.863+3A>G<br>c.863+5G>A	579	419
	GCK_Ex7R_SPL	CATCTCTCACAGGGGCAGGTCATG		c.863+182				
	GCK_Ex8F_SPL	GCTCTGGCTCATTAACGAGGGAAAGA	8	c.864-176	exon 8 (-1)<br>exon 8 (-1)<br>intron 8	c.1019G>A<br>c.1019G>C<br>c.1019+20G>A	786	391
	GCK_Ex8R_SPL	GCCTTGGGGTTGTGAGTGATGT		c.1020-279				
	GCK_Ex9F_SPL	TAGAGGGGGCAGTACTAACCAGTCC	9	c.1020-230	intron 9	c.1190_1253+11dup	647	469
	GCK_Ex9R_SPL	CTCCCACCTCATCCTCCACATTCAT		c.1253-34				
	HNF1A_Ex2F_SPL	CCTACCATCCATCCACCAGT	2	c.327-481	intron 2	c.526+5G>A	866	435
	HNF1A_Ex2R_SPL	GTCTACTCCCGTCCCACAGA		c.526+185				
	HNF1A_Ex3F_SPL	CTTGGGTTGGTAGGAAAGCA	3	c.527-245	intron 3	c.713+10C>T	830	422
	HNF1A_Ex3R_SPL	ATGAAGGGGAGGGTTCTGAG		c.714-60				
	HNF1A_ex4F_SPL	TGTCCAGTTGCCGAGAACTC	4	c.713+91	intron 4	c.955+5G>C	844	477
	HNF1A_ex4R_SPL	GGAATGGGGTTAATTGTGGTGG		c.955+235				
	HNF1A_Ex7F_SPL	ATGACTTGCCAGAGCCACTT	7	c.1310-180	exon 7 (-1)<br>intron 7<br>intron 7	c.1501G>A<br>c.1501+4A>G<br>c.1501+5G>C	604	427
	HNF1A_Ex7R_SPL	TACACACCCAGACACGCACT		c.1501+232				
	HNF1A_Ex8F_SPL	AGCTGAGCAGTTCCCTGTAATG	8-9	c.1502-202	exon 8 (-1)<br>exon 9	c.1623G>A<br>c.1742_1768+2delins	798	502
	HNF1A_Ex9R_SPL	CAGTCCAGTCCCTGAGATGTTC		c.1768+236				
	HNF4A_Ex3F_SPL	ACTCAGCTCTAACACCAACCAG	3	c.225-268	intron 2	c.225-3C>A	577	330
	HNF4A_Ex3R_SPL	AGAGTGGGAGCTTATCTCTCTGA		c.319+214				
	HNF4A_Ex4F_SPL	GCTGATGGGTGGATCACTCTTA	4	c.320-234	exon 4 (-1)	c.426G>A	600	342
	HNF4A_Ex4R_SPL	CAGTGAAGGTGAAGACTCTGCT		c.426+259				
	HNF4A_Ex8F_SPL	TCTGCCTGTGTCTAGGAAATCA	8	c.827-186	exon 8 (-1)	c.1063G>C	600	472
	HNF4A_Ex8R_SPL	GACCATTGCCTTGTTCCCAT		c.1063+177				
sequencing of minigene inserts	pCAS-Seq-F	GGGGTCAATAGCAGTGAGAGG						
	pCAS-Seq-R	GCTCCATTTCACAGGTAGAGA						
RT-PCR and sequencing of RT-PCR products	pCAS-KOI-F	TGACGTCGCCGCCCATCAC						
	pCAS-2R	ATTGGTTGTTGAGTTGGTTGTC						

the forward primers used for PCR all begin with: AAGAAGTGCAGGATC; and all reverse primers begin with: TCAAAACAAGACGCG (complementary sequence to the pCAS2 plasmid necessary for the insertion of the PCR product in the linearized vector via InFusion cloning kit.)

For the exons 5 and 6 of *GCK* and exons 8 and 9 of *HNF1A* , those short and closely located exons were cloned together in the vector.

Table S2: Bioinformatics predictions of splicing alterations for the 36 variants selected for minigene assays on *GCK*, *HNF1A* and *HNF4A* and comparison with experimental data.

Variant location refers to the exon/intron structure of the genes with the indication of the nearest natural splice site (ss). The distance of the exonic variants relative to the nearest reference splice site is indicated between parentheses. Bioinformatics analyses with two algorithms (SpliceAI and SPiP) were performed by using the following decision thresholds, as recommended in the literature: ≥0.2 for SpliceAI [21] and "Alter" regardless the splice score for SPiP [23]. The results of SpliceAI are shown as follow: AG, acceptor gain; AL, acceptor loss; DG, donor gain; DL, donor loss. The value of the delta score (DS) is the difference between the raw score (RS) of the splice site in reference context and the RS of the splice site in variant context. The number in brackets corresponds to the distance of the splice site from the variant tested (the value is positive if the splice site is downstream and negative is the splice site is upstream from the variant tested). SpliceAI indicates for each category the position with the higher DS.

The column "location of cryptic/de novo 3'ss or 5'ss with gain score ≥ 0.2 corresponds to the splice site potentially activated or created that have been predicted by SpliceAI (corresponding to the numbers into brackets).

One limitation in the interpretation of SpliceAI is that this algorithm only gives the position of the cryptic splice site with the higher DS. Therefore, the column "other splice site(s) visualized by SpliceAI Visual" contains two types of information provided by SpliceAI-visual: 1/ additional cryptic splice sites with lower DS and 2/ cryptic splice sites with non-significant DS (i.e. <0.2) but with RS equivalent or superior to that of the consensual splice site in variant context. These 2 types of cryptic splice sites are not provided in first intention by SpliceAI but could be of potential interest.

					SpliceAI 1.3 (± 500 bp from the variant of interest)						
Gène	**Genomic nomenclature hg19**	**Variant location**		**Nucleotide change**	**3'ss gain** AG	3'ss loss AL	**5'ss gain** DG	5'ss loss DL	Location of cryptic/de novo 3'ss or 5'ss with gain scores ≥ 0.2	Other splice site(s) visualized by SpliceAI Visual	Interpretation
GCK	g.44192901T>C	exon 2 (-2)	5'ss	c.207A>G, p.(Ser69=)	0 (-405)	0,07 (157)	**0,54 (-11)**	**0,41 (-1)**	c.208+10		Alteration of the consensus splice site
GCK	g.44192900C>T	exon 2 (-1)	5'ss	c.208G>A, p.(Glu70Lys)	0 (-31)	0,06 (158)	**0,58 (-10)**	**0,95 (0)**	c.208+10		Alteration of the consensus splice site
GCK	g.44192900C>G	exon 2 (-1)	5'ss	c.208G>C, p.(Glu70Gln)	0 (-31)	0,07 (158)	**0,59 (-10)**	**0,98 (0)**	c.208+10		Alteration of the consensus splice site
GCK	g.44192885G>C	intron 2	5'ss	c.208+15C>G	0,02 (173)	0 (5)	**0,6 (5)**	**0,27 (15)**	c.208+10		Creation of a new splice site
GCK	g.44191877G>C	exon 3 (-8)	5'ss	c.356C>G, p.(Ala119Gly)	0 (101)	0 (85)	**0,82 (1)**	0,03 (-7)	c.355		Creation of a new splice site + Alteration of
GCK	g.44190556T>C	exon 4 (-2)	5'ss	c.482A>G, p.(Lys161Arg)	0,01 (145)	0,01 (118)	**0,83 (23)**	**0,48 (-1)**	c.459		Alteration of the consensus splice site + Alt
GCK	g.44189669_44189674del	intron 4	3'ss	c.484-11_484-6del	0,03 (67)	**0,64 (-5)**	0 (65)	**0,67 (-100)**			Alteration of the consensus splice site
GCK	g.44189663C>T	exon 5 (+1)	3'ss	c.484G>A, p.(Gly162Ser)	0,01 (-2)	0,02 (0)	0 (70)	0,04 (-95)			Alteration of the consensus splice site
GCK	g.44189568C>A	exon 5 (-1)	5'ss	c.579G>T, p.(Gly193=)	0,01 (95)	0 (-83)	**0,99 (2)**	**0,55 (0)**	c.577		Alteration of the consensus splice site + Cre
GCK	g.44189564del	intron 5	5'ss	c.579+4del	0 (-175)	**0,48 (100)**	0 (44)	**0,92 (5)**			Alteration of the consensus splice site
GCK	g.44189467A>C	intron 5	3'ss	c.580-9T>G	**0,29 (18)**	0,06 (-9)	0,01 (-92)	0,01 (101)	c.580-27		Alteration of the consensus splice site
GCK	g.44189461G>T	intron 5	3'ss	c.580-3C>A	**0,37 (24)**	**0,65 (-3)**	0,03 (-86)	0,03 (107)	c.580-27		Alteration of the consensus splice site
GCK	g.44189461del	intron 5	3'ss	c.580-3del	**0,38 (25)**	**0,92 (-2)**	0 (125)	0,06 (-85)	c.580-27		Alteration of the consensus splice site
GCK	g.44189361A>C	exon 6 (-3)	5'ss	c.677T>G, p.(Val226Gly)	0 (-62)	0 (124)	**0,31 (14)**	0,1 (2)	c.663		NTR
GCK	g.44189359C>T	exon 6 (-1)	5'ss	c.679G>A, p.(Gly227Ser)	0 (126)	0 (-120)	**0,88 (4)**	**0,63 (0)**	c.675		Alteration of the consensus splice site + Cr
GCK	g.44189359C>G	exon 6 (-1)	5'ss	c.679G>C, p.(Gly227Arg)	0,01 (118)	0,02 (126)	**0,57 (4)**	**0,6 (0)**	c.675		Alteration of the consensus splice site + Cr
GCK	g.44189354C>T	intron 6	5'ss	c.679+5G>A	0 (123)	0,01 (131)	**0,38 (21)**	**0,26 (5)**	c.663		Alteration of the consensus splice site
GCK	g.44187447G>T	intron 6	3'ss	c.680-15C>A	**0,58 (-2)**	0,18 (-15)	0 (-477)	0,01 (-188)	c.680-13		Alteration of the polypyrimidine tract
GCK	g.44187438G>C	intron 6	3'ss	c.680-6C>G	**0,99 (-1)**	**0,45 (-6)**	0,01 (-498)	0,01 (-189)	c.680-5		Alteration of the consensus splice site + Cre
GCK	g.44187246C>T	intron 7	5'ss	c.863+3A>G	0 (-215)	0 (-234)	0,08 (-130)	**0,23 (3)**		c.853	Alteration of the consensus splice site
GCK	g.44187244C>T	intron 7	5'ss	c.863+5G>A	0 (-208)	0,02 (-232)	0,09 (53)	**0,81 (5)**		c.853	Alteration of the consensus splice site
GCK	g.44186062C>T	exon 8 (-1)	5'ss	c.1019G>A, p.(Ser340Asn)	0,01 (95)	0 (-482)	**0,44 (-17)**	**0,94 (0)**	c.1019+17	c.1011	Alteration of the consensus splice site
GCK	g.44186062C>G	exon 8 (-1)	5'ss	c.1019G>C, p.(Ser340Thr)	0,01 (95)	0 (-482)	**0,45 (-17)**	**0,93 (0)**	c.1019+17	c.1011	Alteration of the consensus splice site
GCK	g.44186042C>T	intron 8	5'ss	c.1019+20G>A	0 (115)	0,01 (350)	**0,53 (3)**	**0,39 (28)**	c.1019+17		Creation of a new splice site
GCK	g.44185085_44185159dup	exon 9 - intron 9	5'ss	c.1190_1253+11dup	0,01 (394)	0,11 (-109)	0,15 (-14)	0,01 (1885)			Alter by SPiCE
HNF1A	g.121426840G>A	intron 2	5'ss	c.526+5G>A	0 (-477)	0,02 (-295)	**0,65 (-37)**	**0,92 (-5)**	c.494		Alteration of the consensus splice site
HNF1A	g.121431519C>T	intron 3	5'ss	c.713+10C>T	0 (485)	0,01 (-196)	0,05 (-2)	0,02 (-10)			Creation of a new splice site
HNF1A	g.121432213G>C	intron 4	5'ss	c.955+5G>C	0 (94)	0,04 (-209)	**0,74 (2)**	**0,74 (-5)**	c.955+7		Alteration of the consensus splice site + Cre
HNF1A	g.121435468G>A	exon 7 (-1)	5'ss	c.1501G>A, p.(Ala501Thr)	0 (25)	0,01 (-174)	**0,8 (42)**	0,08 (0)	c.1501+42		Alteration of the consensus splice site + Alte
HNF1A	g.121435472A>G	intron 7	5'ss	c.1501+4A>G	0 (-4)	0,01 (-178)	**0,83 (38)**	0,12 (-4)	c.1501+42		Alteration of the consensus splice site
HNF1A	g.121435473G>C	intron 7	5'ss	c.1501+5G>C	0 (20)	0,01 (-179)	**0,92 (37)**	**0,64 (-5)**	c.1501+42		Alteration of the consensus splice site
HNF1A	g.121437192G>A	exon 8 (-1)	5'ss	c.1623G>A, p.(Gln541=)	0 (6)	**0,2 (73)**	**0,24 (5)**	0,01 (0)	c.1623+5		Alteration of the consensus splice site
HNF1A	g.121437404_121437432delinsACAGGG	exon 9 - intron 9	5'ss	c.1742_1768+2delinsACAGGG	0	0,06 (0)	0,04	1(0)			Alteration of the consensus splice site
HNF4A	g.43036018C>A	intron 2	3'ss	c.225-3C>A	0,02 (-187)	**0,51 (3)**	0 (-7)	0,12 (97)		c.225-10	NTR
HNF4A	g.43042440G>A	exon 4 (-1)	5'ss	c.426G>A, p.(Gln142=)	0 (8)	**0,32 (-106)**	**0,29 (8)**	**0,79 (0)**	c.426+8	c.411	Alteration of the consensus splice site
HNF4A	g.43052894G>C	exon 8 (-1)	5'ss	c.1063G>C, p.(Gly355Arg)	0 (-15)	0,17 (-236)	**0,21 (65)**	**0,94 (0)**	c.1063+65		Alteration of the consensus splice site

SPIP 2.1			Pangolin 1.0.2			AbSplice-DNA (Max_score)	Final interpretation of bioinformatics predictions				Observed in minigene assay		
InterConfident	SPiP score	Position of the strongest cryptic splice site	significant values	Location of diminished splicing sites	Location of increased splicing sites		**SpliceAI**	**SPIP**	**Pangolin**	**AbSplice-DNA**	3'ss **gain**	5'ss **gain**	minigene
98.41 % [91.47 % - 99.96 %]	0,986		-11:0.49|-1:-0.67	c.208	c.208+10	0,11	1	1	1	1		c.208+10	Intronic retention (10 bp)
98.41 % [91.47 % - 99.96 %]	0,994		-10:0.63|0:-0.82|	c.208	c.208+10	0,31	1	1	1	1		c.208+10	Intronic retention (10 bp)
98.41 % [91.47 % - 99.96 %]	0,994		-10:0.61|0:-0.83	c.208	c.208+10	0,36	1	1	1	1		c.208+10	Intronic retention (10 bp)
47.89 % [39.44 % - 56.42 %]	0,422	c.208+10	5:0.77|15:-0.5	c.208	c.208+10	0,31	1	1	1	1		c.208+10	Intronic retention (10 bp)
69.33 % [61.29 % - 76.59 %]	0,602	c.355	1:0.57		c.355	0,031	1	1	1	1			No splicing defect
100 % [97.32 % - 100 %]	0,858		23:0.6|-1:-0.61	c.483	c.459	0,2	1	1	1	1		c.459	Exon skipping + exonic deletion (24 bp)
98.41 % [91.47 % - 99.96 %]	0,922		-100:-0.62|-5:-0.72			not available	1	1	1	NA			In-frame exon skipping
35.81 % [28.11 % - 44.1 %]	0,304					0,0037	0	1	0	0			No splicing defect
98.41 % [91.47 % - 99.96 %]	0,938	c.577	2:0.83|0:-0.76	c.579	c.577	0,32	1	1	1	1		c.577	Exonic deletion (2 bp)
98.41 % [91.47 % - 99.96 %]	0,956		5:-0.76|100:-0.62	c.579		not available	1	1	1	NA			In-frame exon skipping
23.61 % [16.94 % - 31.4 %]	0,134		18:0.49|-9:-0.31	c.580	c.580-27	0,034	1	1	1	1	c.580-27		Complex alteration
98.41 % [91.47 % - 99.96 %]	0,972		24:0.62|-3:-0.8	c.580	c.580-27	0,19	1	1	1	1	c.580-27		Complex alteration
98.41 % [91.47 % - 99.96 %]	0,988		25:0.62|-2:-0.85	c.580	c.580-27	not available	1	1	1	NA	c.580-27		Complex alteration
09.3 % [02.59 % - 22.14 %]	0,082		14:0.2		c.663	0,031	1	0	1	1		c.663	Exonic deletion (16bp)
85.91 % [79.27 % - 91.06 %]	0,764	c.675	4:0.78|0:-0.8	c.679	c.675	0,42	1	1	1	1		c.663 / c.675	Complex alteration
96.71 % [92.49 % - 98.92 %]	0,768	c.675	4:0.39|16:0.38|0:-0.75	c.679	c.675 and c.663	0,14	1	1	1	1		c.663 / c.675	Complex alteration
98.41 % [91.47 % - 99.96 %]	0,942		21:0.39|5:-0.5	c.679	c.663	0,095	1	1	1	1		c.663	Exonic deletion (16bp)
98.41 % [91.47 % - 99.96 %]	0,94	c.680-13	-2:0.36|-15:-0.54	c.680	c.680-13	0,05	1	1	1	1	c.680-13		Exon skipping + intronic retention (13 bp)
98.41 % [91.47 % - 99.96 %]	0,968	c.680-5	-1:0.77|-6:-0.76	c.680	c.680-5	0,22	1	1	1	1	c.680-5		Intronic retention (5 bp)
100% [97.32-100%]	0,854		13:0.35|3:-0.56	c.863	c.853	0,006	1	1	1	0		c.853	Exonic deletion (10 bp)
98.41 % [91.47 % - 99.96 %]	0,998		15:0.4|5:-0.66	c.863	c.853	0,32	1	1	1	1		c.853	Exonic deletion (10 bp)
98.41 % [91.47 % - 99.96 %]	0,926	c.1011 / c.1019+2	-17:0.47|8:0.37|0:-0.69	c.1019	c.1019+17 and c.101	0,35	1	1	1	1		c.1011 / c.1019+17	Complex alteration
98.41 % [91.47 % - 99.96 %]	0,928	c.1011 / c.1019+2	-17:0.48|8:0.39|0:-0.69	c.1019	c.1019+17 and c.101	0,34	1	1	1	1		c.1011 / c.1019+17	Complex alteration
10.53 % [06.14 % - 16.53 %]	0,116	c.1019+17	3:0.71|20:-0.48	c.1019	c.1019+17	0,041	1	1	1	1		c.1019+17	Intronic retention (17 bp)
98.41 % [91.47 % - 99.96 %]			0:0.43			not available	1	1	1	NA			Intronic retention (75 bp)
98.41 % [91.47 % - 99.96 %]	1		-37:0.68|-5:-0.81	c.526	c.494	0,31	1	1	1	1		c.494	Exonic deletion (32 bp)
47.89 % [39.44 % - 56.42 %]	0,43	c.713+8				0,0029	0	1	0	0			No splicing defect
98.41 % [91.47 % - 99.96 %]	0,946	c.955+7	2:0.67|-5:-0.78	c.955	c.955+7	0,25	1	1	1	1		c.955+7	Exon skipping + intronic retention (7 bp)
98.41 % [91.47 % - 99.96 %]	0,954		42:0.49|0:-0.58	c.1501	c.1501+42	0,22	1	1	1	1			In-frame exon skipping
85.91 % [79.27 % - 91.06 %]	0,738		38:0.55|-4:-0.6	c.1501	c.1501+42	0,19	1	1	1	1		c.1501+42	Exon skipping + intronic retention (42 bp)
96.71 % [92.49 % - 98.92 %]	0,798		37:0.66|-5:-0.77	c.1501	c.1501+42	0,23	1	1	1	1		c.1501+42	Exon skipping + intronic retention (42 bp)
54 % [45.68 % - 62.16 %]	0,502	c.1623+5				0,089	1	1	0	1			Out-of-frame exon skipping
98.41 % [91.47 % - 99.96 %]			NA			not available	1	1	NA	NA			Out-of-frame exon skipping
01.85 % [00.38 % - 05.32 %]	0,018		3:-0.4	c.225		0,15	1	0	1	1	c.225-10		Intronic retention (10 bp)
98.41 % [91.47 % - 99.96 %]	1		-15:0.21		c.411	0,26	1	1	1	1		c.411	Exon skipping + exonic deletion (15 bp)
98.41 % [91.47 % - 99.96 %]	0,99	c.1063+4	0:-0.79	c.1063		0,3	1	1	1	1		c.882 / c.1063+65	Complex alteration

Table S3: Classification of variants tested in minigene assay. For each variant is indicated the ACMG criteria we applied and the classification prior to minigene assay based on the information available in the lab. The conclusion of the minigene assay and the predicted effect on the protein are indicated. In case of multiple transcripts, the clinical impact of mis-splicing defects being difficult to predict, we have modulated the PS3 criterion to moderate evidence (PS3_M) of mis-splicing as suggested by the SpliceACORD consortium [27].

				Prior to minigene assay						After minigene assay		
Gene	Variant location	(position)	Nucleotide change, predicted protein effect	ACMG criteria	ACMG classification	Minigene assay		mRNA changes	Predicted protein change	Added criteria for each alteration	ACMG criteria	ACMG classification
GCK	exon2 (-2)	Donor	c.207A>G, p.(Ser69=)	PM2 PP3 PP4	VUS	Intronic retention (10 bp)	▼E2q10	r.207_209delinsCGGTACCACATG	p.Glu70Glyfs*4	+ PS3	PS3 PM2 PP4	LP
GCK	exon 2 (-1)	Donor	c.208G>A, p.(Glu70Lys)	PS4 PM2 PP1 PP2 PP3 PP4	P	Intronic retention (10 bp)	▼E2q10	r.208_209delinsAGTACCACATG	p.Glu70Serfs*4	+ PS3	PS3 PS4 PM2 PP1 PP4	P
GCK	exon 2 (-1)	Donor	c.208G>C, p.(Glu70Gln)	PM2 PP2 PP3 PP4	VUS	Intronic retention (10 bp)	▼E2q10	r.208_209delinsCGTACCACATG	p.Glu70Argfs*4	+ PS3	PS3 PM2 PP4	LP
GCK	intron 2	Donor	c.208+15C>G	PM2 PP3 PP4	VUS	Intronic retention (10 bp)	▼E2q10	r.208_209insGTACCACATG	p.Glu70Glyfs*4	+ PS3	PS3 PM2 PP4	LP
GCK	exon 3 (-8)	Donor	c.356C>G, p.(Ala119Gly)	PM2 PP2 PP3	VUS	No splicing defect					PM2 PP2 PP3	VUS
GCK	exon 4 (-2)	Donor	c.482A>G, p.(Lys161Arg)	PM2 PP2 PP3	VUS	Exon skipping + exonic deletion (24 bp)	△E4q24<br>**△E4**	r.556_579del<br>r.364_483del	p.Val154_Lys161del<br>p.Leu122_Lys161del	+ PM4<br>+ PS3	PS3 PM2	LP
GCK	intron 4	Acceptor	c.484-11_484-6del	PM2 PP3 PP4	VUS	In-frame exon skipping	△E5	r.484_579del	p.Gly162_Gly193del	+ PS3	PS3 PM2 PP4	LP
GCK	exon 5 (+1)	Acceptor	c.484G>A, p.(Gly162Ser)	PM2 PP2 PP3 PP4	VUS	No splicing defect					PM2 PP2 PP3 PP4	VUS
GCK	exon 5 (-1)	Donor	c.579G>T, p.(Gly193=)	PM2 PP3	VUS	Exonic deletion (2 bp)	△E5q2	r.578_579del	p.Asp194Leufs*2	+ PS3	PS3 PM2	LP
GCK	intron 5	Donor	c.579+4del	PM2 PP1 PP3 PP4	VUS	In-frame exon skipping	△E5	r.484_579del	p.Gly162_Gly193del	+ PS3	PS3 PM2 PP1 PP4	LP
GCK	intron 5	Acceptor	c.580-9T>G	PM2 PP3 PP4	VUS	Complex alteration	WT<br>▼E5q109<br>▼E6p27<br>△E5▼E6p27	r.579_580ins109<br>r.579_580ins27<br>r.484_579delins27	p.Asp194Valfs*39<br>p.Gly193_Asp194ins(9)<br>p.Gly162_Gly193delins(9)	+ PS3<br>+ PM4<br>+ PM1	PS3_M PM2 PP4	VUS
GCK	intron 5	Acceptor	c.580-3C>A	PM2 PP3 PP4	VUS	Complex alteration	WT<br>▼E5q109<br>▼E6p27<br>△E5▼E6p27	r.579_580ins109<br>r.579_580ins27<br>r.484_579delins27	p.Asp194Valfs*39<br>p.Gly193_Asp194ins(9)<br>p.Gly162_Gly193delins(9)	+ PS3<br>+ PM4<br>+ PM1	PS3_M PM2 PP4	VUS
GCK	intron 5	Acceptor	c.580-3del	PM2 PP3 PP4	VUS	Complex alteration	WT<br>▼E5q108<br>▼E6p26<br>△E5▼E6p26	r.579_580ins109<br>r.579_580ins26<br>r.484_579delins26	p.Gly193_Asp194ins(36)<br>p.Asp194Hisfs*19<br>p.Gly162Hisfs*19	+ PM4<br>+ PS3<br>+ PS3	PS3_M PM2 PP4	VUS
GCK	exon 6 (-3)	Donor	c.677T>G, p.(Val226Gly)	PM2 PM5 PP2 PP3	LP	Exonic deletion (16bp)	WT<br>△E6q16	r.663_679del	p.Val222Alafs*67	+ PS3	PS3_M PM2 PM5 PP2	LP
GCK	exon 6 (-1)	Donor	c.679G>A, p.(Gly227Ser)	PS4 PM2 PP1 PP2 PP3 PP4	P	Complex alteration	WT<br>△E6q16<br>△E6q4	r.663_679del<br>r.676_679del	p.Val222Alafs*67<br>p.Val226Alafs*67	+ PS3<br>+ PS3	PS3_M PS4 PM2 PP1 PP4	P
GCK	exon 6 (-1)	Donor	c.679G>C, p.(Gly227Arg)	PM2 PM5 PP2 PP3 PP4	LP	Complex alteration	WT<br>△E6q16<br>△E6q4	r.663_679del<br>r.676_679del	p.Val222Alafs*67<br>p.Val226Alafs*67	+ PS3<br>+ PS3	PS3_M PM2 PM5 PP4	LP
GCK	intron 6	Donor	c.679+5G>A	PM2 PP1 PP3 PP4	VUS	Exonic deletion (16bp)	WT<br>△E6q16	r.663_679del	p.Val222Alafs*67	+ PS3	PS3_M PM2 PP1 PP4	LP
GCK	intron 6	Acceptor	c.680-15C>A	PM2 PP3 PP4	VUS	Exon skipping + intronic retention (13 bp)	▼E7p13<br>△E7	r.679_680ins13<br>r.680_863del	p.Gly227Aspfs*52<br>p.Thr228Metfst*5	+ PS3	PS3 PM2 PP4	LP
GCK	intron 6	Acceptor	c.680-6C>G	PS2 PM2 PP1 PP3 PP4	LP	Intronic retention (5 bp)	▼E7p5	r.679_680ins5	p.Gly227Alafs*69	+ PS3	PS2 PS3 PM2 PP1 PP4	P
GCK	intron 7	Donor	c.863+3A>G	PM2 PP3 PP4	VUS	Exonic deletion (10 bp)	△E7q10	r.854_863del	p.Gln286Metfs*5	+ PS3	PS3 PM2 PP4	LP
GCK	intron 7	Donor	c.863+5G>A	PM2 PP3 PP4	VUS	Exonic deletion (10 bp)	△E7q10	r.854_863del	p.Gln286Metfs*5	+ PS3	PS3 PM2 PP4	LP
GCK	exon 8 (-1)	Donor	c.1019G>A, p.(Ser340Asn)	PM2 PP2 PP3 PP4	VUS	Complex alteration	△E8q8<br>▼E8q17<br>△E8	r.1012_1019del<br>r.1019_1020delins18<br>r.864_1019del	p.Val338Argfs*118<br>p.Ser340Lysfs*19<br>p.Tyr289_Ser340del	+ PS3<br>+ PS3<br>+ PS3	PS3 PM2 PP4	LP
GCK	exon 8 (-1)	Donor	c.1019G>C, p.(Ser340Thr)	PM2 PP2 PP3 PP4	VUS	Complex alteration	△E8q8<br>▼E8q17<br>△E8	r.1012_1019del<br>r.1019_1020delins18<br>r.864_1019del	p.Val338Argfs*118<br>p.Ser340Thrfs*19<br>p.Tyr289_Ser340del	+ PS3<br>+ PS3<br>+ PS3	PS3 PM2 PP4	LP
GCK	intron 8	Donor	c.1019+20G>A	PM2 PP1 PP3 PP4	VUS	Intronic retention (17 bp)	▼E8q17	r.1019_1020ins17	p.Ser340Argfs*19	+ PS3	PS3 PM2 PP1 PP4	LP
GCK	exon 9 - intron	Donor	c.1190_1253+11dup	PM2 PP3 PP4	VUS	Intronic retention (75 bp)	▼E9q75	r.1253_1254ins75	p.Ser418Argfs*2	+ PS3	PS3 PM2 PP4	LP
HNF1A	intron 2	Donor	c.526+5G>A	PM2 PP1 PP3 PP4	VUS	Exonic deletion (32 bp)	△E2q32	r.495_526del	p.Trp165*	+ PS3	PS3 PM2 PP1 PP4	LP
HNF1A	intron 3	Donor	c.713+10C>T	PP3	VUS	No splicing defect					BS3	VUS
HNF1A	intron 4	Donor	c.955+5G>C	PM2 PP3	VUS	Exon skipping + intronic retention (7 bp)	▼E4q7<br>△E4	r.955_956ins7<br>r.714_955del	p.Val320Lysfs*12<br>p.Ala239Cysfs*10	+ PS3	PS3 PM2	LP
HNF1A	exon 7 (-1)	Donor	c.1501G>A, p.(Ala501Thr)	PP1 PP3 PP4	VUS	In-frame exon skipping	△E7	r.1310_1501del	p.Gly437_His500del	+ PS3	PS3 PP1 PP4	LP
HNF1A	intron 7	Donor	c.1501+4A>G	PM2 PP3 PP4	VUS	Exon skipping + intronic retention (42 bp)	▼E7q42<br>**△E7**	r.1501_1502ins42<br>r.1310_1501del	p.His500_Ala501ins(14)<br>p.Gly437_His500del	+ PM4<br>+ PS3	PS3 PM2 PP4	LP
HNF1A	intron 7	Donor	c.1501+5G>C	PM2 PP3	VUS	Exon skipping + intronic retention (42 bp)	▼E7q42<br>**△E7**	r.1501_1502ins42<br>r.1310_1501del	p.His500_Ala501ins(14)<br>p.Gly437_His500del	+ PM4<br>+ PS3	PS3 PM2	LP
HNF1A	exon 8 (-1)	Donor	c.1623G>A, p.(Gln541=)	PM2 PP3	VUS	Out-of-frame exon skipping	△E8	r.1502_1623del	p.Ala501Glyfs*7	+ PS3	PS3 PM2	LP
HNF1A	exon 9 - Intron	Donor	c.1742_1768+2delinsACAGGG	PM2 PM4 PP3	VUS	Out-of-frame exon skipping	△E9<br>△E8E9	r.1624_1768del<br>r.1502_1768del	p.Val542Lysfs*70<br>p.Ala501_Thr589del	+ PS3	PS3 PM2	LP
HNF4A	intron 2	Acceptor	c.225-3C>A	PM2 PP3	VUS	Intronic retention (10 bp)	**WT**<br>▼E3p10	r.224_225ins10	p.Arg75Serfs*6	+ PS3	PS3_M PM2	VUS
HNF4A	exon 4 (-1)	Donor	c.426G>A, p.(Gln142=)	PM2 PP3 PP4	VUS	Exon skipping + exonic deletion (15 bp)	WT<br>△E4q15<br>△E4	r.412_426del<br>r.320_426del	p.Ala136_Gln142del<br>p.Ala107Aspfs*25	+ PM4<br>+ PS3	PS3_M PM2 PP4	VUS
HNF4A	exon 8 (-1)	Donor	c.1063G>C, p.(Gly355Arg)	PM2 PP3	VUS	Complex alteration	▼E8q65<br>△E8q181	r.1063_1064delins66<br>r.883_1063del	p.Gly355Argfs*39<br>p.Val295Glyfs*17	+ PS3<br>+ PS3	PS3 PM2	LP
